# Predicting dwell fatigue life in titanium alloys using modelling and experiment

**DOI:** 10.1038/s41467-020-19470-w

**Published:** 2020-11-17

**Authors:** Yilun Xu, Sudha Joseph, Phani Karamched, Kate Fox, David Rugg, Fionn P. E. Dunne, David Dye

**Affiliations:** 1 Department of Materials, Imperial College, London, SW7 2AZ UK; 2 Department of Materials, University of Oxford, Oxford, OX1 3PJ UK; 3 Rolls-Royce plc, Derby, DE24 8BJ UK

**Keywords:** Engineering, Materials science

## Abstract

Fatigue is a difficult multi-scale modelling problem nucleating from localised plasticity at the scale of dislocations and microstructure with significant engineering safety implications. Cold dwell fatigue is a phenomenon in titanium where stress holds at moderate temperatures lead to substantial reductions in cyclic life, and has been implicated in service failures. Using discrete dislocation plasticity modelling complemented by transmission electron microscopy, we successfully predict lifetimes for ‘worst case’ microstructures representative of jet engine spin tests. Fatigue loading above a threshold stress is found to produce slip in soft grains, leading to strong dislocation pile-ups at boundaries with hard grains. Pile-up stresses generated are high enough to nucleate hard grain basal dislocations, as observed experimentally. Reduction of applied cyclic load alongside a temperature excursion during the cycle lead to much lower densities of prism dislocations in soft grains and, sometimes, the elimination of basal dislocations in hard grains altogether.

## Abstract (web summary)

Material fatigue is the most common source behind failures of mechanical structures. Here the authors combine transmission electron microscopy, high-resolution electron backscatter diffraction and discrete dislocation plasticity modeling to study the underlying mechanism of dwell fatigue in titanium alloys.

## Introduction

Fatigue was famously implicated in the Comet jetliner failures in the 1950s, and these and other accidents spurred research into the phenomenon and the use of fatigue resistance as a fundamental design parameter^1^. Often occurring well below the nominal yield stress, the fatigue strength, or stress for a given run-out cyclic life, then becomes the design parameter for a structural material in the absence of a pre-existing notch or other stress concentration feature. For high integrity defect-free titanium alloys, bearing steels or powder metallurgy nickel superalloys, crack nucleation^2^ occurs at the length scale of the microstructure, e.g. a carbide or twin boundary in a superalloy, or a grain boundary between crystals of strongly contrasting orientation in a titanium alloy. However this then leads to a sampling problem if the initiating microstructural feature occurs infrequently in the material. Low-volume test pieces may not contain the critical microstructural feature, such that higher fatigue lives result. More critically, failure may occur by mechanisms that are different to those occurring in service in high-volume components such that a statistical analysis of test piece failures may not, in itself, resolve this problem. The importance of extreme statistics in microstructure has been addressed by Ozturk et al.^3^. In addition, a frequent problem in the field has been that the stresses that have to be applied in laboratory testing to generate failure are >90% of the yield stress^4^, whereas in component spinning rig tests, dwell failures can be obtained at only around 70% of yield, which leads to a concern as to whether the mechanisms in test piece testing are in fact representative of those in service. This, we suggest, may also be an artefact of the stressed volume of material subjected to testing and the likelihood of a test piece containing the critical microstructural feature.

Cold dwell fatigue occurs in some *α*-Ti alloys^5,6^ in which local creep deformation may occur in grains well orientated for basal or prism slip leading to stress redistribution onto grains badly orientated for slip (*c*-axis parallel to loading direction). For the latter, while pyramidal slip is possible to activate, the critical resolved shear stresses are considerably higher than those for basal or prism slip activation. The stress redistribution, which occurs during the dwell period, is often referred to as load shedding. A very considerable reduction (>5×, termed the ‘dwell-debit’^7^) in the number of cycles to failure may occur relative to conventional low cycle fatigue testing. Aero-engine in-service operating conditions lead to thermal transients in addition to mechanical loading, giving rise to the potential thermal alleviation of dwell fatigue^8^.

Macrozones, also called micro-textured regions (MTRs)^9^, are clusters of contiguous grains^10^ with similar crystallographic orientation which are inherited from the growth of *α* colonies in Ti alloys^11^ in processing. These can be problematic as dislocations can glide easily across grain boundaries sharing high crystallographic commonality^12^, producing pile-ups at the boundary between ‘soft’ macrozones comprising grains well-oriented for slip and ‘hard’ macrozones of grains poorly oriented for slip^13^. Bantounas et al.^14^ and Zhang et al.^15,16^ found that macrozones with their *c*-axes oriented close to loading direction are favourable for facet crack nucleation^17,18^. Creep is known to develop in soft macrozones^19^ by virtue of dislocation escape from obstacles, and glide during the dwell period giving rise to stress redistribution (also termed ‘load shedding’^20^) to the adjacent hard regions, eventually leading to faceted crack nucleation on the irrational planes often inclined 10−20° with respect to (0001) in hard grains^21,22^.

Transmission electron microscopy (TEM) investigations on dislocation interactions across the grain boundaries near crack initiation under dwell fatigue have been carried out previously^4,23–25^. Extensive glide in Ti alloys is responsible for creep^26^ and subsequent load shedding at room temperature. We have recently studied the dislocation interactions at soft/hard grain boundaries under both low cycle^27,28^ and dwell fatigue^28^. 〈a〉-prism pile-ups in the soft grain were observed to nucleate 〈a〉-dislocations in the hard grain. The latter were found to multiply by source activation via cross slip and junction formation. This leads to a higher dislocation density in the hard grain under dwell fatigue than in conventional low cycle fatigue.

Discrete dislocation plasticity (DDP) explicitly simulates the activities of dislocations, whose collective motion along defined slip planes gives rise to plasticity within crystalline metals. The conventional two-dimensional DDP framework used^29^ has been previously applied to study localised micro-deformation under various loading conditions, including tension^30^, micro-pillar compression^31^, bending^32^, indentation^33^ and sliding^34^. Zheng et al.^35^ proposed a rate-sensitive DDP model that incorporated thermally activated escape of pinned dislocations, thereby allowing for creep, which has been employed to investigate load shedding^20,36,37^ in titanium alloys. By incorporating property temperature-dependence and thermal strain, Xu et al.^38^ developed a temperature-enhanced DDP framework which has been applied to understand the thermomechanical alleviation (TMA) in Ti-834 alloys under anisothermal conditions.

In this work, we use an integrated experimental and numerical methodology to study dwell fatigue in alloy Ti-834 with TEM, high-resolution electron backscatter diffraction (HR-EBSD) and DDP modelling. The objective is to understand the underpinning mechanisms that explain the relative cyclic rates of strain accumulation observed for isothermal and thermally alleviated cyclic plasticity in Ti-834, and to provide the mechanistic basis for the dwell fatigue and the dwell debit. We deliberately create both samples and models containing the worst-case scenario microstructure feature, that of adjacent ‘hard’ and ‘soft’ macrozones, in order to avoid the sampling problem discussed above. It should be noted that this approach can only be employed because we have an a priori hypothesis as to the underlying mechanism, which we seek here to test. We demonstrate that prism slip occurs in soft grains, leading to dislocation pile-up at hard grain boundaries and so to a stress concentration at the hard−soft grain boundary, provided the applied stress exceeds a threshold of approximately 0.80*σ*_*y*_ in Ti-834. The consequent prism dislocation pile-up stresses in the soft grain drive basal dislocation nucleation in the adjacent hard grain. The role of the ‘dwell’ within the dwell fatigue loading cycle is revealed to drive cyclic creep accumulation^39^ in the soft grain by prism dislocation activation, thermally activated escape and pile-up at hard grain boundaries, in turn leading to progressive cyclically increasing stress on the hard grains. Good agreement between the HR-EBSD, TEM and fatigue testing experiments and the modelling is obtained.

## Results

### Fatigue tests

**Fig. 1:**
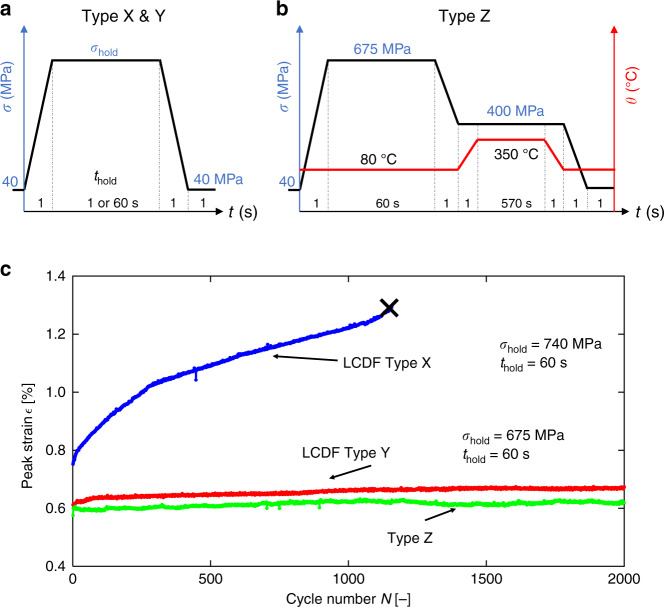
Cyclic strain evolutions for thermomechanical fatigue cycling. **a**, **b** Stress and temperature fatigue loading types examined. **c** Evolution of peak strain in each cycle for the three loading types. Thermal strain has been subtracted from the total strain in Loading Type Z and thus only mechanical strains are shown. The sample subjected to dwell loading Type X failed at 1157 cycles, denoted by the black cross.

Fatigue test cycles were conducted at 80 °C with peak stresses up to 740 MPa, well below the yield stress (~845 MPa). The hold time at stress was either 1 s (low cycle fatigue, LCF) or 60 s for dwell fatigue (low cycle dwell fatigue, LCDF); we focus in particular on LCDF with peak stresses of 740 MPa (dwell Loading Type X) and 675 MPa (Loading Type Y). A thermal alleviation test (Loading Type Z) was also examined where the stress was reduced to 400 MPa and the temperature then increased to 350 °C, Fig. 1. Figure 1c shows the evolution of the peak strain in each cycle; conventional dwell fatigue at 740 MPa (Type X) evolves as observed previously (e.g. ref. ^40^) where cyclic creep strain accumulation occurs during the stress holds. A lower peak stress of 675 MPa, Type Y, produces a much lower cyclic strain accumulation rate. Inclusion of the elevated temperature secondary stress hold, Type Z, results in the lowest rate of evolution of macroscopic strain of the three cycles. Therefore, a thermal alleviation effect^41^ is observed, with reduced overall creep strain accumulation in Loading Type Z than in Type Y.

### TEM observation

Dislocation analysis was carried out on the TEM foils taken from the gauge section of the failed samples in bright field (BF) imaging mode. Each grain examined was tilted to at least three different beam directions **B** and three different **g** vectors under two-beam conditions in order to analyse the dislocations. Scanning transmission electron microscopy (STEM) was used to capture the overall dislocation structures in a grain, with the grain tilted to one of its zone axes.

**Fig. 2:**
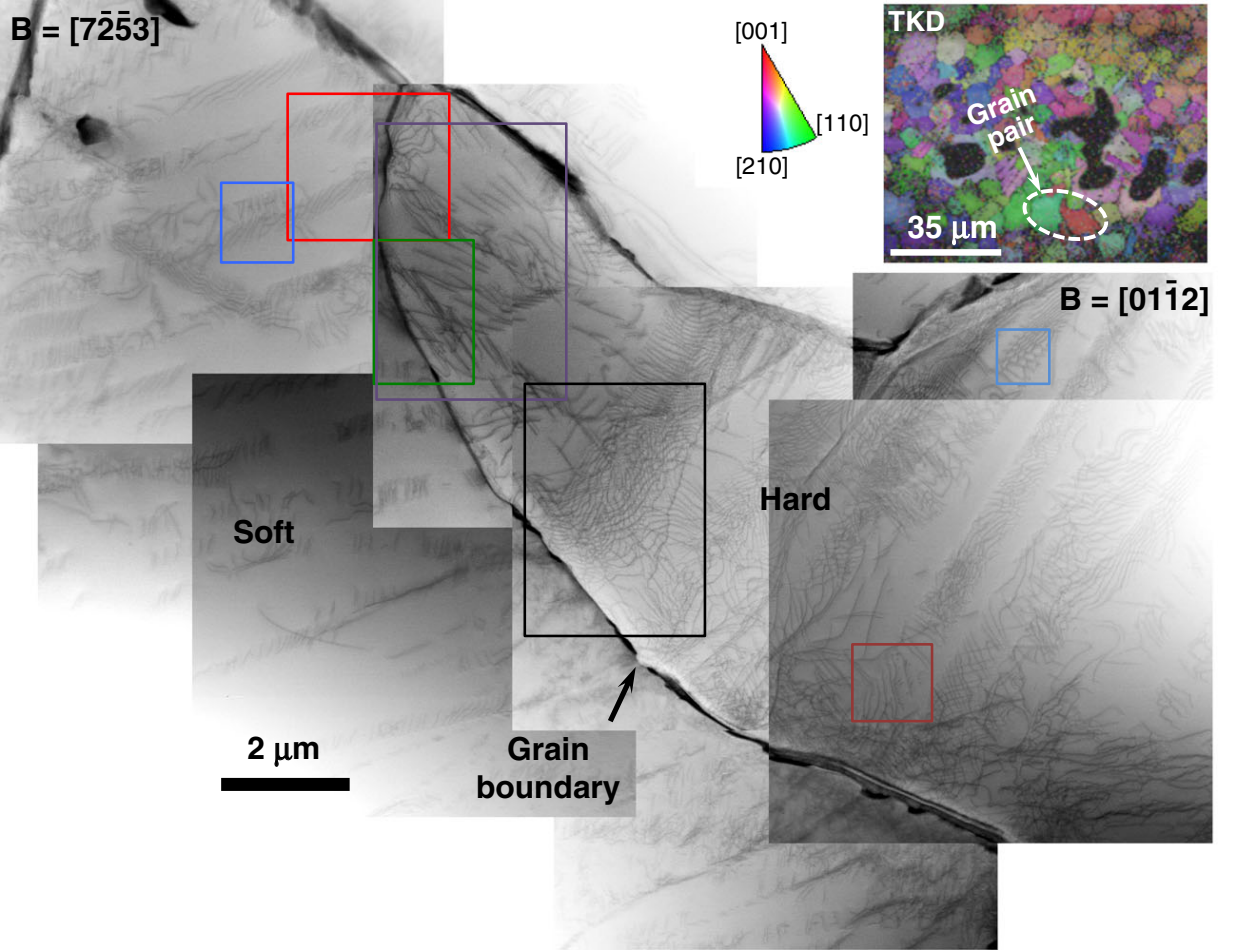
STEM composite micrograph showing the dislocation structures under Loading Type X. Structures observed in the soft/hard grain pair of the sample under dwell Loading Type X with beam direction $\left[ {7\bar 2\bar 53} \right]$ for soft grain and $\left[ {01\bar 12} \right]$ for hard grain. TKD (inset) shows the analysed grain pair orientation with respect to the loading direction; the loading direction is normal to the foil. The highlighted regions are examined further in Fig. 3.

Soft/hard crystallographically oriented grain pairs were selected for analysis utilising TKD on the TEM foils. The inverse pole figure (IPF) map in the inset of Fig. 2 highlights the grain pair investigated. The soft and hard grains have their *c*-axes oriented ≈81.5° and 11° to the loading direction respectively. The grain pair with this maximum possible misorientation has been chosen to ensure that the resistance to slip transmission across the boundary is maximum so that the dislocation mechanisms due to load shedding could be visualised. The BF-STEM composite micrograph in Fig. 2 shows the overall dislocation structures observed in this grain pair of the sample under Loading Type X with the soft grain tilted to ${\mathbf{B}} = \left[ {7\bar 2\bar 53} \right]$ and the hard grain then tilted to ${\mathbf{B}} = \left[ {01\bar 12} \right]$. BF-STEM imaging permits observation of all the dislocations simultaneously, except for those with line directions parallel to the beam, and is relatively insensitive to bend contours and other imaging artefacts. A number of dislocation pile-ups were seen both in soft and hard grains with higher dislocation density in the hard grain. Nucleation of dislocations in the hard grain occurred where the soft grain pile-ups meet the hard grain.

**Fig. 3:**
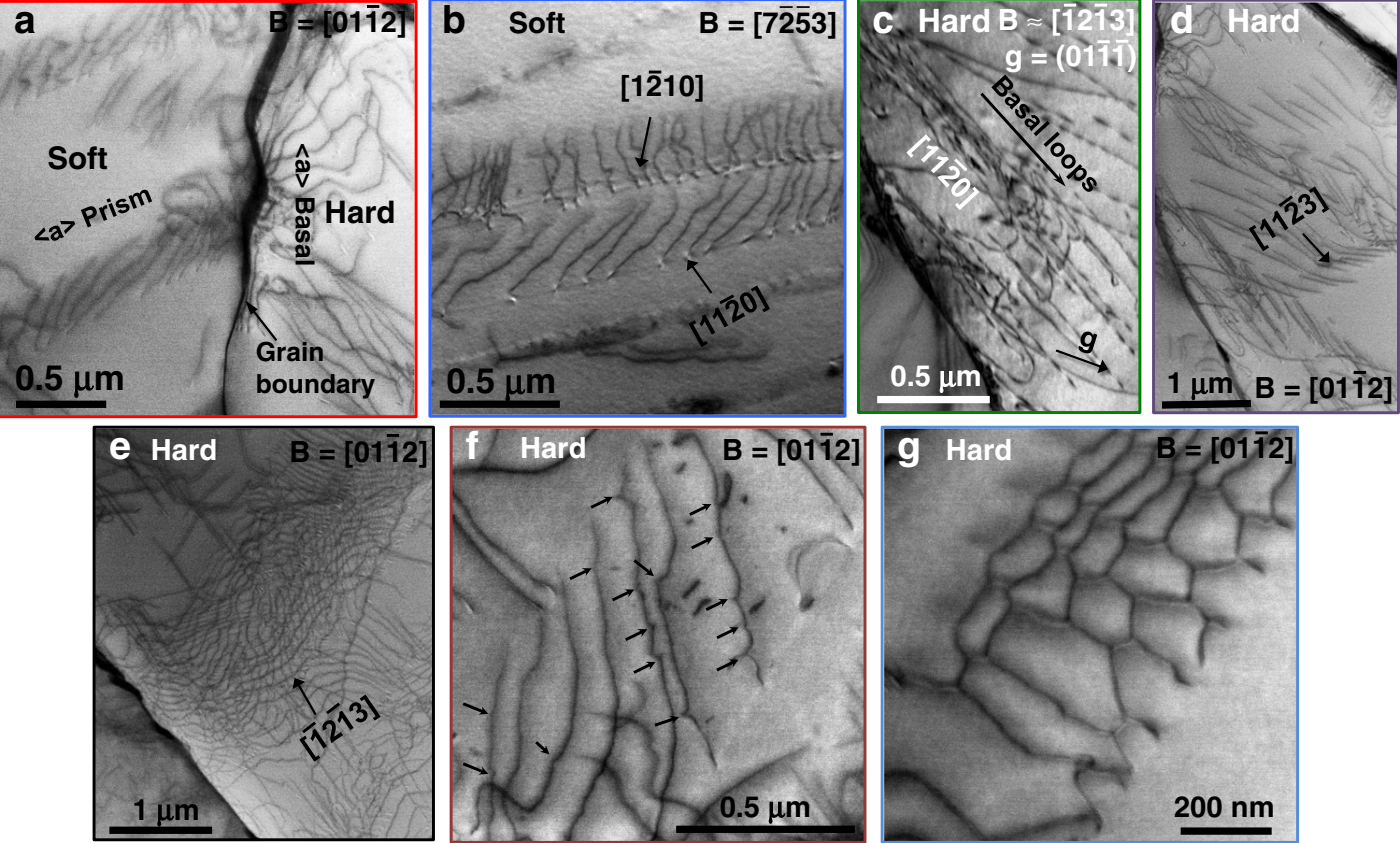
Basal, prism and pyramidal dislocations and pile-ups in soft/hard grain combination. High magnification images showing **a** the nucleation of *a* basal dislocations in hard grain by the impingement of prism pile-ups in soft grain, **b** two kinds of *a* prism pile-ups in the soft grain, **c** nucleation of basal loops from the boundary. **d**, **e** *c* + *a* dislocations in the hard grain. **f** *c* + *a* dislocation cross-slip and **g** a *c* + *a* dislocation network. All images were captured in STEM mode except (**c**), which is a BF-TEM image under two-beam condition. The beam conditions are shown in each sub-figure.

The dislocation structures in this grain pair are analysed further in Fig. 3. The high magnification image in Fig. 3a shows the nucleation of dislocations in a hard grain by the impingement of pile-ups in the soft grain. These are 〈a〉-prism pile-ups causing nucleation of 〈a〉-basal dislocations in the hard grain. Two kinds of prism pile-ups were observed in the soft grain, $\left[ {11\bar 20} \right]\left( {1\bar 100} \right)$ and $\left[ {1\bar 210} \right]\left( {\bar 1010} \right)$ (Fig. 3b), where the former is the primary slip system. The basal dislocations in the hard grain, of $\left[ {11\bar 20} \right]$ type, were found to nucleate as dislocation loops from the boundary, Fig. 3c. The 〈c + a〉 dislocation pile-ups in the hard grain were found to be of $\left[ {11\bar 23} \right]$ $\left( {\bar 1011} \right)$ type, Fig. 3d. The other long 〈c + a〉 dislocations are $\left[ {\bar 12\bar 13} \right]$ type, Fig. 3e. Cross-slip of 〈c + a〉 dislocations was observed (shown by arrows), with some debris in the form of dislocation loops, Fig. 3f. In addition, some 〈c + a〉 dislocation networks, frequently left over after annealing and which can act as sources^42^, were observed in the hard grain, Fig. 3g.

**Fig. 4:**
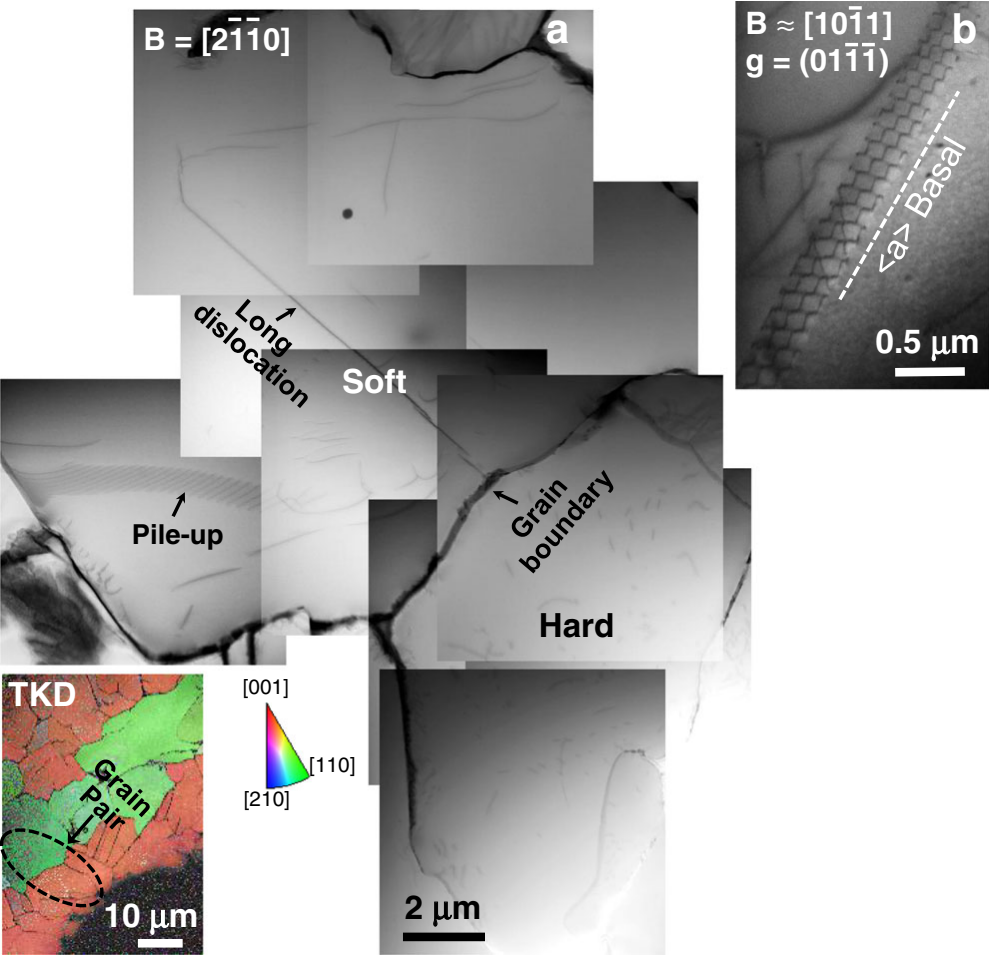
Dislocation structures observed in the soft/hard grain pair for dwell Loading Type Y. **a** STEM composite micrograph showing the dislocation structures observed in the soft/hard grain pair when the soft grain is tilted to beam direction $\left[ {2\overline {11} 0} \right]$ of the sample, under Loading Type Y. The TKD in the inset shows the analysed grain pair orientation with respect to the loading direction and **b** BF-TEM in two-beam condition showing a basal dislocation network in soft grain.

The STEM composite micrograph in Fig. 4a shows the dislocation structures observed in the soft/hard grain pair for dwell Loading Type Y. TKD, inset, on the TEM foil shows the grain pair of interest. The soft and hard grains had *c*-axes oriented ≈87° and 14.5° to the loading direction, respectively. Very few dislocations were observed in this grain pair, in contrast to the previous pair for dwell Type X. A long dislocation line and a pile-up were observed in the soft grain, where the pile-up did not impinge upon the hard grain of interest; therefore, that pile-up is not analysed. A few random dislocation lines were observed in the hard grain. The long dislocation line in the soft grain consisted of a network of dislocations under two beam conditions ${\mathbf{B}} = \left[ {10\bar 11} \right]$ and ${\mathbf{g}} \approx \left[ {01\overline {11} } \right],$ Fig. 4b. ***g***.***b*** invisibility analysis shows that the network has all three types of *a* dislocations, gliding on basal planes. It was in the edge-on condition under ${\mathbf{B}} = \left[ {2\overline {11} 0} \right]$ in Fig. 4a. Such dislocation networks are often found in as-received materials and are believed to form during processing^42^. The dislocations observed in the hard grain were of 〈c + a〉-type.

### DDP analysis

Temperature-coupled DDP calculations were performed for 100 cycles for all three load types, in order to investigate further the dependency of the cyclic behaviour on load cycle, the dislocation structures formed and the local stress states established; detailed comparisons with the experimental TEM and HR-EBSD observations are made.

**Fig. 5:**
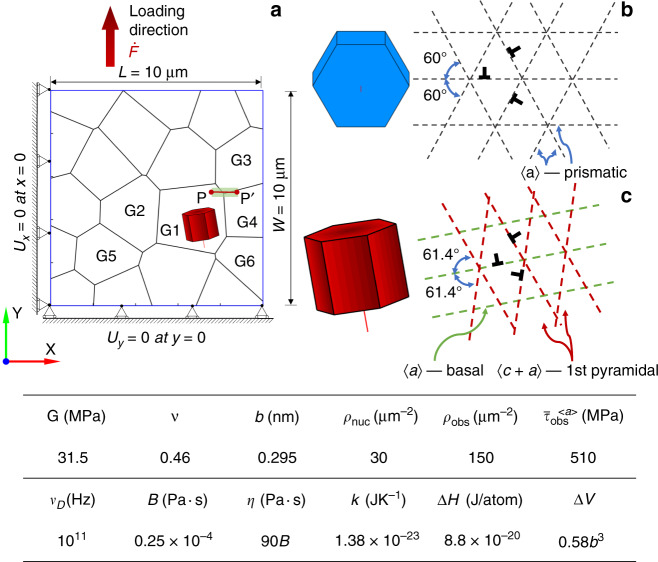
Discrete dislocation modelling of hard/soft orientation grains in Ti-IMI834. **a** Polycrystalline 2D discrete dislocation plasticity model for alloy Ti-834 sample that is loaded in the *y*-direction. Schematic diagrams of slip system configuration in HCP **b** soft grains containing three 〈a〉-prismatic slip systems, and **c** hard grains containing one 〈a〉-basal plus two 〈c + a〉-pyramidal slip systems. The inset table tabulates DDP model material parameters for Ti-834 at *θ* = 80 °C.

A 2D polycrystal model with 20 *α*-HCP grains in a 10 μm × 10 μm region was utilised for DDP modelling of the strongly textured Ti-834 sample, Fig. 5a. The microstructure was generated using VGRAIN Voronoi software^43^. Force-controlled-loading was applied in the loading direction, *y*. Displacement (*U*) constraints were imposed on the left-hand-side (i.e. *U*_*x*_ = 0 along *x* = 0) and bottom (i.e. *U*_*y*_ = 0 along *y* = 0) boundaries. Analyses of differing loading conditions (e.g. sinusoidal stress variation with 120 MPa amplitude at peak load) were carried out in order to establish local sensitivity to microstructure-influenced mechanical states, and were found to be small. The plane strain constraint was achieved by confining slip activation in HCP grains to three 〈a〉-prismatic slip systems in soft grains, two first-order 〈c+a〉-pyramidal and one 〈a〉-basal slip system in hard grains, the configurations of which are schematically illustrated in Fig. 5b, c respectively, inspired by Rice^44^. The orientation of the hard grain with respect to loading direction is chosen to match the experiment (see later) at 11°. The spacing between each set of parallel slip planes was set to be 100*b*. The microstructure of the 2D DDP model aims to represent the observed hard−soft grain orientation combinations shown in the set EBSD map of Fig. 2. More specifically, one hard grain, whose *c*-axis was oriented at 11° with respect to the loading direction (*y*-axis), was included in the 2D polycrystal model and labelled as G1 in Fig. 5a. The surrounding soft grains (*c*-axis out of plane) were assigned crystal orientations from the EBSD map in Fig. 2 and hence the model represents the microstructure of the strongly textured Ti-834 sample. The beta phase was not explicitly modelled. Soft grains G2−G6 were used to investigate the local strain, stress and dislocation structures developed, for comparison to experiment. The stress distribution along path P−P′ (Fig. 5a) was assessed at different instants in the loading to investigate stress concentration and load shedding^37^ near the triple junction of grains G1, G3 and G4. The polycrystal sample domain was uniformly discretised into 10^4^ first-order bilinear finite elements to achieve a sufficient spatial resolution of local deformation. The material properties and their temperature sensitivity for alloy Ti-834 have previously been determined from experimental data^38^ and their values at *θ* = 80 °C are given in the inset table, Fig. 5c. The temperature-dependent nucleation strength (considering the slip strength of pyramidal slip used was triple that of basal and prismatic slip^45^, i.e. $\bar \tau _{{\mathrm{{nuc}}}}^{ \langle c + a \rangle } = 3\bar \tau _{{\mathrm{{nuc}}}}^{ \langle a \rangle }$) along with the density of dislocation sources and obstacles were calibrated against macroscopic stress−strain response curves of polycrystalline Ti-834 samples at temperatures ranging from room temperature to 400 °C. The rate-sensitivity parameters (i.e. Δ*H* and Δ*V*) were obtained by matching the stress relaxation curves at two different strain loading rates at *θ* = 80 °C. These parameters were validated by comparison with the cyclic strain response of Ti-834 polycrystal samples.

**Fig. 6:**
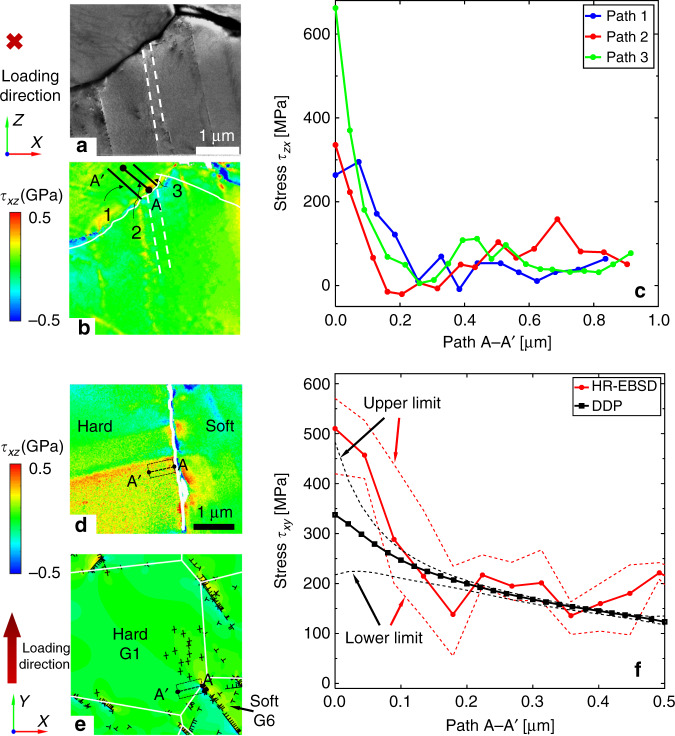
HR-EBSD measured and DDP predicted stresses at hard/soft orientation grains. **a** Forescatter detector image showing the soft/hard grain boundary of the sample under dwell Loading Type X where the HR-EBSD scan was performed with a step size 0.02 μm, **b** HR-EBSD shear stress distribution in the same region, and **c** the line scans of the HR-EBSD shear stress in the hard grain generated by the pile-up. The line scans are marked in (**b**). Note these stresses lie within a plane normal to the loading direction. In-plane shear stress near the soft/hard grain boundary under dwell Type X obtained by **d** HR-EBSD in a plane parallel to the loading direction after failure at 1157 cycles, and **e** the DDP model after 100 cycles. Grain boundaries are denoted by white lines. The in-plane shear stresses in the hard grain obtained from HR-EBSD and DDP modelling are compared in (**f**), where the path scans and the region of interest are marked in (**d**, **e**), respectively.

The residual stress state measured near the soft/hard grain boundary of the sample under dwell Loading Type X measured by HR-EBSD is shown in Fig. 6. The image in the SEM was obtained by retracting the EBSD detector, such that the forescattered (ARGUS TM) diodes on the system were at a position directly below the sample and a bright field image could be obtained, Fig. 6a. The shear stress *τ*_*xz*_ (i.e. within the plane perpendicular to the loading direction) distribution calculated by this method was elevated near the boundary where the dislocation pile-up impinges the boundary, Fig. 6b. Figure 6c shows the variation of the shear stress along the three paths depicted in Fig. 6b, which decreases approximately as a function of one-over square root of distance from the grain boundary^46,47^. HR-EBSD measurement of intragranular strain requires selection of a reference point from which strain differences are determined (the ‘d0 problem’). It is noted that the measured stresses in Fig. 6c drop away to near-zero with distance from the grain boundary suggesting that the stress magnitudes determined are sensible. Shear stresses (*τ*_*xy*_) within a plane lying parallel to the loading direction and at a hard−soft grain boundary have also been extracted from the sample subject to dwell Type X using HR-EBSD. This was modelled with DDP utilising the polycrystal model, Fig. 5a; while this is not an exact replication of grain morphology, the hard−soft crystallographies and grain boundary with respect to the loading direction are faithfully reproduced in the model. The HR-EBSD results are shown in Fig. 6d, and the corresponding DDP-predicted dislocation distribution is shown in Fig. 6e. A shear stress concentration near the grain boundary was observed from both HR-EBSD measurements in Fig. 6d and DDP analysis in Fig. 6e due to the 〈a〉-prismatic dislocation pile-ups in the adjacent soft grain. Quantitative comparison of the in-plane shear stresses obtained from HR-EBSD and DDP analysis is shown in Fig. 6f for paths A−A′ shown in Fig. 6d, e, respectively. The average shear stresses (black squares) along ten paths parallel to path A−A′ and within the rectangle in the DDP contour plot (Fig. 6e) decrease from the dislocation pile-up at the grain boundary to the hard grain interior, which is consistent with and reasonably close to the trend obtained from the HR-EBSD measurement (red circles). The upper and lower limits of the stresses measured along the paths by HR-EBSD and predicted in DDP are also reported in red and black dashed lines, respectively. The measured stresses are obtained after 1157 loading cycles compared with (for reasons of computational time) 100 cycles for the DDP analysis, so that somewhat higher stresses are anticipated from the experimental measurement.

**Fig. 7:**
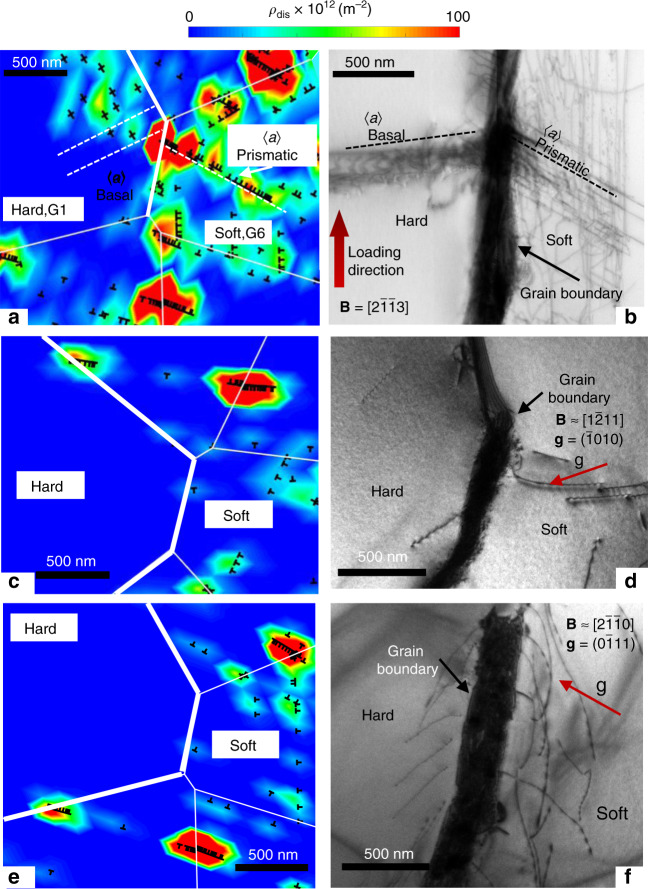
Dislocation structures from DDP modelling and TEM characterisation. **a** The hard-soft grain boundary under dwell Loading Type X from discrete dislocation analysis after 100 cycles and **b** the corresponding TEM composite image after sample failure at 1157 cycles. The predicted total dislocation density distribution is plotted in (**a**). Dislocation structures near the hard-soft grain boundary under Loading Type Y (**c**, **d**) and Type Z (**e**, **f**) obtained from **c**, **e** discrete dislocation analysis after 100 cycles and **d**, **f** TEM observation after 2000 cycles. The total dislocation density distribution is also plotted in (**a**, **c**, **e**). Note that (**c**) is vertically flipped to visually match the grain boundary and hence compare to the dislocation structure observed in the corresponding TEM observation (**d**).

A one-to-one comparison of the dislocation structure predicted by the DDP analysis with TEM examination on the plane parallel to the loading direction under dwell Loading Type X is shown in Fig. 7. Strong 〈a〉-prismatic dislocation activity within the soft grain leads to pile-up towards the soft/hard grain boundary in both the TEM and DDP analyses in dwell Type X, with the highest applied peak stress. 〈a〉-basal dislocations are nucleated in the hard grain due to the stress states established by 〈a〉-prismatic dislocation pile-ups in the soft grain. The predicted 〈a〉-basal dislocation pile-up in the hard grain is not as intense as that observed from the TEM but 〈a〉-basal dislocations are nonetheless predicted to nucleate within the hard grain, as observed in the TEM. The lower predicted basal dislocation density in the DDP model may result from the small number of cycles (i.e. 100) simulated, but the overall dislocation structure and pile-up pattern obtained from the DDP analysis is nonetheless consistent with the TEM examination. A qualitative comparison of the dislocation structures from DDP analysis (loading direction in plane) and TEM examination (loading direction out of plane) under Types Y and Z is shown in Fig. 7c–f. However, the DDP results in Fig. 7c, e and the TEM observations in Fig. 7d, f for Loading Type Y and Z both show that the dislocation structures that result indicate a low dislocation density in the hard grain and a significantly lower density and distribution of dislocations in the soft grain under Type Y and Z compared to those for dwell Type X, Fig. 7a. More dislocations, though not strongly piling-up, are observed for Type Z than for Type Y in both the DDP analysis and TEM observations. The multiplication of dislocations under Type Z is anticipated to be a consequence of the decrease of dislocation nucleation strength during the temperature excursions.

**Fig. 8:**
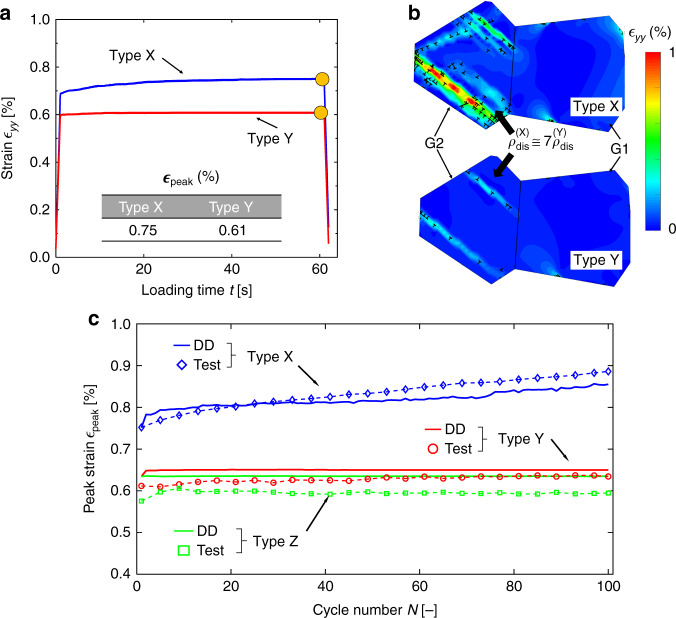
Discrete dislocation plasticity predicted and measured strains under cyclic loading. Comparison of **a** macroscopic strain evolution in the first cycle and **b** local strain variation and the superimposed dislocation structures for dwell Loading Type X and Y. The strain contours are shown at the unloaded instant within the hard (G1)/soft (G2) grain pair in Fig. 5a. **c** Peak strain against cycles for the three loading cycles predicted by DDP model (solid lines) and from experiment (symbols). The strains contain the mechanical contributions only, i.e. thermal strains have been subtracted.

The macroscopic (average) strain evolution during the first cycle under dwell Loading Type X and Type Y are predicted using the DDP polycrystal model and shown in Fig. 8a. During the stress hold after initial elastic loading, (creep) strain continues to increase to peak values of 0.75 and 0.61%. The local strain variations and corresponding dislocation structure within soft/hard grain pair G1−G2, see Fig. 5a, are compared for Type X and Y in Fig. 8b at the unload point in the cycle, Fig. 8a. Many more *a*-prismatic dislocations are nucleated and maintained in the soft grain (G2) under Type X (seven times that under Type Y), and their collective motion along slip planes results in the higher macroscopic strain shown in Fig. 8a. Intense dislocation pile-ups are observed for Type X near the soft/hard grain boundary, generating strain in the adjacent hard grain, G1. The dislocation pile-ups in Type Y do not appear as strong due to the lower peak stress and thus result in less dislocation activity. While Type Z (see Fig. 1) includes the thermal excursion to higher temperature during the second (reduced) stress hold, this is not found to lead to any additional creep strain evolution. In fact, on the contrary, the thermal excursion is found to a small extent to reduce the cyclic strain accumulation and its predicted distribution within the soft grain is found to be similar to but smaller than that for Type Y, Fig. 8b. The single cycle strain analyses from the DDP model under the three loading types have been extended to cyclic behaviour over 100 cycles. The cyclic peak strain evolution for the three cycles predicted by the DDP model (solid lines) are compared with measured behaviour from the fatigue tests (symbols) in Fig. 8c. From the DDP results, the cyclic peak strain was found to accumulate rapidly for Type X, with a total increase of 0.1% in 100 cycles, which is considerably higher than for Types Y and Z because of the lower peak stress in Types Y and Z and the additional thermal alleviation occurring in Type Z, respectively. The latter in Type Z approximately counteracts the (very small) positive strain accumulated in the primary hold period. Hence, the resulting net rate of increase of peak strain in th is loading is near zero (~0.001% in 100 cycles) such that overall ${\mathrm{d}}\varepsilon ^X/{\mathrm{d}}N \gg {\mathrm{d}}\varepsilon ^Y/{\mathrm{d}}N > {\mathrm{d}}\varepsilon ^Z/{\mathrm{d}}N$. The ranking of the rate of strain accumulation predicted by the DD model is in qualitative and quantitative agreement with the experimental measurements, and suggests that cyclic dwell Loading Type X would lead to earlier failure. The accumulated strain difference arising between Loading Types Y and Z in experiment appears to be marginally larger than in the DD prediction. We attribute this to sample-to-sample variations in the initial sample state (i.e. initial dislocation structure^33^ and thermal residual stress).

**Fig. 9:**
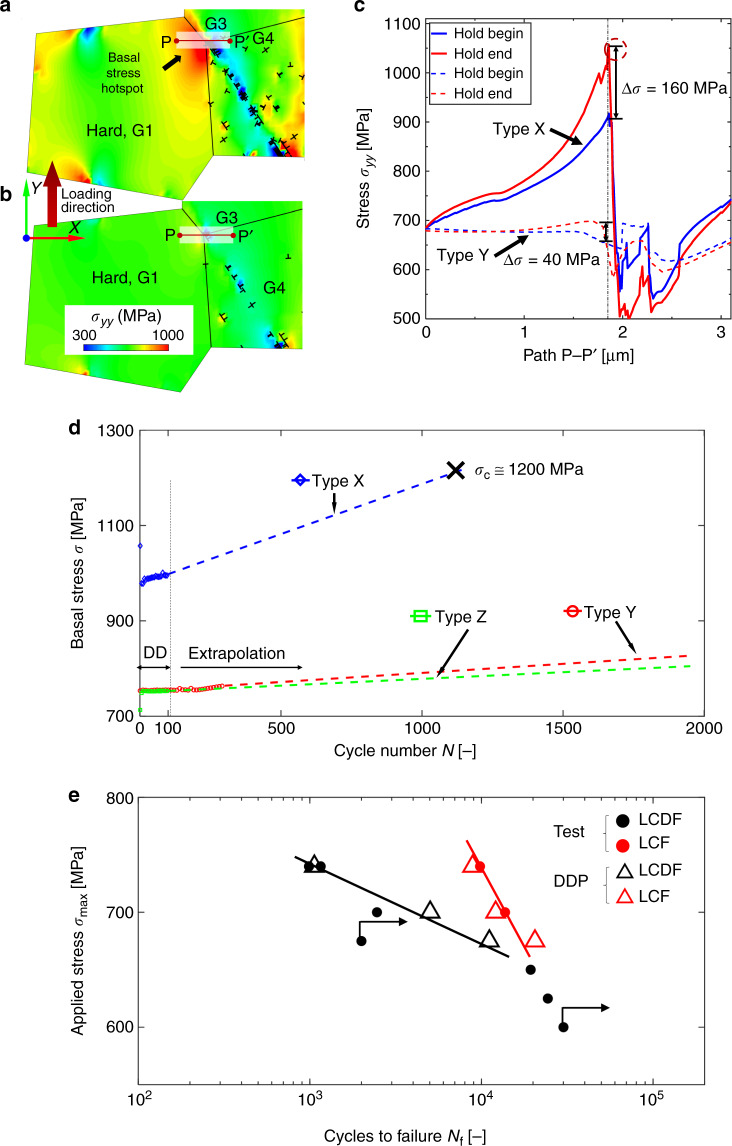
Cyclic stresses, and DDP predicted and experimentally measured cycles to failure. Localised DDP yy-stress distribution at the triple junction of grains G1−G3−G4 after the stress hold for dwell loading **a** Type X and **b** Type Y. **c** Comparison of load shedding (averaged over the grey regions) along the path P−P′ (shown in (**a**)) before and after the stress hold. **d** DDP predicted basal stresses for Type X (the sample failed at 1157 cycles), Y and Z based on the DDP simulation for 100 calculated cycles, together with extrapolated stress data. (For cycle Y, DDP calculations for 300 cycles enabled the error resulting from extrapolation to be estimated as ~4%.) **e** Predicted (DDP model) and experimental (test) *S*−*N* curves under low cycle fatigue (LCF) and low cycle dwell fatigue (LCDF) with various applied peak stresses. The up-right arrow indicates a run-out test i.e. sample did not fail within 30,048 LCDF cycles at 600 MPa, or 2000 LCDF cycles at 675 MPa.

The yy-stress distribution and instantaneous dislocation structure at the end of the stress hold for dwell Loading Types X and Y are illustrated in Fig. 9a, b, respectively, showing a location near the triple junction made by grains G1, G3 and G4 (defined in Fig. 5a). A basal stress hot spot is established in Type X by virtue of a strong 〈a〉-prismatic dislocation pile-up in the soft grains (i.e. G3 and G4) and towards the soft/hard grain boundaries. In contrast, for loading Type Y, there is no evident basal stress concentration near the triple junction because of the low applied stress and hence weak dislocation pile-up. Average yy-stresses along 20 paths parallel to P−P′ and within the shaded rectangle containing the triple junction in Fig. 9a, b are calculated. The stresses before (in blue) and after (in red) the remote stress hold for the first cycle of Type X (solid line) and Type Y (dashed line) are reported in Fig. 9c. Much stronger load shedding is observed in Type X with stress redistribution of Δ*σ* = 160 MPa onto the hard grain at the grain boundary whereas that in Type Y shows a much lower value of Δ*σ* = 40 MPa. The significant stress redistribution from soft grains (G3 and G4) to the hard grain (G1) is driven by creep in the soft grains due to thermally activated dislocation escape from obstacles (recall Eq. (2)) under the high applied peak stress hold (at ~0.9*σ*_*y*_) in Type X. The maximum basal stress *σ*_*〈*basal*〉*_ evolutions in the hard grain (G1, Fig. 5a) have been calculated over 100 cycles for loading X, Y and Z and are shown in Fig. 9d. These data are then used to extrapolate the stresses using the stabilised rates predicted by the DDP analysis for each loading type and are shown in Fig. 9d. In previous work^48^, a critical stress (*σ*_c_ = 1200 MPa) for quasi-cleavage facet nucleation, based on the experimentally determined *c*-axis cleavage strength^49^, has been utilised for titanium *α* grains. Therefore, with the knowledge of the per-cycle rate of change of basal stress, it becomes possible to predict the dwell fatigue nucleation life for Loading Type X. Accordingly, this life is determined to be 1062 cycles which is very close to the experimentally observed failure life of 1157 cycles. We suspect that the closeness of this prediction is fortuitous, but it nonetheless demonstrates the predictive capability of the overall modelling approach. In addition, 200 extra cycles were calculated for Type Y (giving 300 calculated cycles in total) such that the error in the DDP calculated stress compared with the extrapolated stress for this loading type was determined, leading to an estimated error in predicted cycles to dwell facet nucleation of ~4%. We note that the model predictions give cycles to dwell crack nucleation and do not account for cycles required to propagate the crack. However, due to the nature of the experimental samples containing a single hard macrozone region of significant size fraction of the cross-section, propagation is anticipated to be rapid (reflected by the short duration strain rate increases measured in the run-up to sample fracture). Hence model crack nucleation predictions at least provide *S*−*N* curve trends. Application of the same failure criterion using the DDP analysis to Loading Types Y and Z predict that these would not result in failure until ~10^4^ cycles. In the experiments, 2000 cycles were imposed without sample failure for these cycles. The same predictive failure criterion is utilised for both LCF and LCDF life for cycle Type X over a range of applied stresses in order to compare with the experimental observations in a classical *S*−*N* curve and are shown in Fig. 9e with remarkable success. In addition to providing good quantitative agreement with experimental lifetimes for both LCF and LCDF loading, the dwell stress threshold below which the dwell debit vanishes is also well captured.

## Discussion

The ‘bad metal sandwich’ samples of titanium alloy Ti-834 in this study comprised the HIPping together of two strongly textured plates from which samples were extracted so as to guarantee the presence of a hard−soft (macrozone) grain pair under uniaxial loading, in turn reflecting conditions that are believed to create a vulnerability to dwell fatigue failure of aero-engine disc components. Under dwell fatigue loading with an applied peak load of ~0.88σ_y_, the bad metal sandwich showed considerable cyclic creep strain. With peak load at or less than ~0.80σ_y_, the cyclic creep strain effectively diminished to zero. The dwell fatigue life for the former was 1157 cycles whereas tests with the latter had not led to failure by 2000 cycles. The hypothesis originally posed by Bache and Evans^4,5^ to explain the observation of facets in hard grains, based upon the elastic solution for stress ahead of a dislocation pile-up^50^, was that slip and dislocation pile-up was anticipated in well-orientated (soft) grains, and that this would lead to pile-up stresses in an adjacent badly orientated (hard) grain of sufficient magnitude so as to nucleate a facet within the hard grain. This hypothesis said nothing explicitly about the role of the dwell part of the cycle, which was first shown by Hasija et al.^20^ to lead to creep in the soft grain and correspondingly to stress redistribution on to the hard grain. Subsequent experimental^18,51^ and numerical work^36,37,52^ showed that the ‘dwell’ within dwell fatigue loading leads to cyclic evolution of creep strain in the soft grain and hence to progressive stress redistribution, in turn giving cyclically increasing stresses in the hard grain and particularly the hard grain boundary region. The absence of the dwell part of the loading eliminates the soft grain creep and therefore the corresponding ratcheting up of hard-grain stress and the associated reduction in lifetime, termed the dwell fatigue debit. The present work examining dwell fatigue on (effectively) hard−soft macrozone samples provides unequivocal evidence that prism slip in the soft grain leads to dislocation pile-up, revealed by TEM, and that this generates high stress concentrations at the prism pile-up within the adjacent hard grain, evidenced by HR-EBSD elastic strain measurement. In turn, these stresses have been shown by DDP modelling of representative microstructures to be sufficiently high to nucleate basal dislocations in the hard grain. The TEM studies in the present work provide the evidential basis for this. Hence, insofar as Bache and Evans’ hypothesis provides some of the mechanistic explanation for dwell fatigue, the present study reinforces this aspect that high pile-up stresses do indeed develop within the hard grain. Our previous analytical calculation predicts that the maximum stress plane would be near basal [≈2.5° to (0002)] under dwell fatigue^28^. In addition, the basal dislocations established within the hard grain may also be crucial for the facet nucleation mechanism.

The DDP model established in this work for Ti-834 similarly shows the nucleation and motion of prism dislocations, and their pile-ups at hard grain boundaries. It also replicates the TEM observations that basal dislocations are nucleated in the adjacent hard grain. In addition, comparison of the DDP-predicted hard grain stress concentration and distribution with ex situ HR-EBSD measurement carried out after sample unloading showed quantitative agreement. Hence the DDP model is utilised to show that the average cyclic (creep) strain evolution is qualitatively and quantitatively captured by the model for the range of thermomechanical loading examined in the experimental programme. Further, the DDP model demonstrates that the role of the ‘dwell’ part of the loading cycle is to induce soft-grain creep by prism dislocation activation and pile-up, which in turn leads to progressive cyclically increasing hard grain boundary stress concentrations and the increase in basal dislocation density in the hard grain. This led to early fatigue failure (the dwell debit) in the test for dwell Loading Type X. If the applied load is reduced, with or without a thermal temperature excursion, the soft grain cyclic creep straining becomes minimal, and the hard grain cyclic stress increase diminishes away. The dwell debit then similarly reduces to zero. Hence we argue that this paper provides underpinning evidence and demonstration of the roles of crystallography (hard−soft grain combination), the dislocation activation and structure, and the ‘dwell’ (driving up hard grain dislocation density) in affecting the life debit in dwell fatigue.

Facet formation may be associated with soft (basal)−hard grain combinations^9,18,22^ as opposed to the soft (prism)−hard system considered in this work, although there is also experimental evidence of soft (prism)−hard combination dwell cracking^22^. Subsequent investigation of the initiating facets in these samples suggest that in the present case, basal slip was implicated in the soft grain, as opposed to the prism slip utilised in the modelling. The occurrence of bas〈a〉 or pri〈a〉 slip in the soft grain appears in the literature to be case-dependent. It has been argued^24^ that bas〈a〉 slip will be more damaging because there are fewer easy secondary slip systems to activate. In either case, the key point is that 〈a〉 slip in the soft grain, piling up at a macrozone boundary in slip bands, is the key step in crack initiation, and that the relative availability of secondary slip systems is what provides the characteristic temperature and time response of cold dwell fatigue. Modelling studies^53^ have been done to investigate both soft grain creep evolution and stress redistribution (load shedding) on to hard grains for the two separate cases of soft (basal) and soft (prism) grains adjacent to a hard grain. This became possible with the extraction of the differing intrinsic basal and prism slip system rate sensitivities from controlled micro-pillar tests^54^. The overall characteristics under dwell fatigue were found to be similar but interestingly, the soft (basal) case led to somewhat higher creep evolution and hence to higher stresses developing on the hard grain. This would support some of the experimental observations made in the literature.

Industrially, it is important that temperature excursions, e.g. during the cruise portion of a flight, can relax the dislocation network and provide a protective effect, as evidenced by the difference in dislocation structures observed in TEM and the reduced strains and hard-grain stresses accumulated in the DDP model. It is also important to note that most lab-based dwell testing of uniaxial fatigue samples requires much higher stresses (>0.9*σ*_*y*_) to generate dwell failures than are observed in spin tests, which we attribute to the relatively small volume of material tested in test piece testing. In the present work this limitation is overcome by deliberately modelling the hypothesised worst-case scenario microstructural feature, and arranging to test material also containing such features. The modelling and experiment then reproduce dwell failures at relatively lower stresses that correspond closely to those observed in spin tests of large disc forgings.

## Methods

**Fig. 10:**
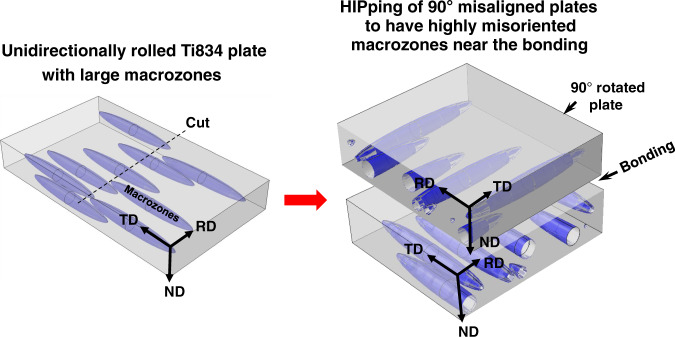
Processing to construct hard/soft macrozone samples. Schematic diagram showing the processing of bonded plate samples with highly misoriented macrozones near the bond line.

Figure 10 illustrates the Ti-834 processing scheme used to intentionally develop strongly textured macrozones. In contrast to standard processing routes, limited super-transus beta breakdown rolling was followed by relatively low temperature unidirectional rolling and globularisation. Two plates with their rolling directions at 90° to each other were then hot isostatically pressed (HIPped) to provide a planar interface with a very high probability of worst case grain-orientation pairs. The HIPped plate was then vacuum solution treated at 1020 °C for 2 h followed by nitrogen gas fan quenching, aging in vacuum at 700 °C for 2 h and air cooling. Samples were then prepared with the planar interface along the RD of the top plate aligned parallel to the loading direction so as to establish a hard/soft texture pairing under load. Fatigue tests were conducted using a 100 kN servohydraulic test frame using cylindrical samples with gauge 9 mm in diameter and 36 mm in length. Test temperature was measured using Optris infrared pyrometers (Model: LT-CF2). Strain measurements were carried out using an optical strain monitoring system and digital camera with in-house software. The measurement system was verified against a calibrated micrometre and validated using conventional extensometry readings. The system was controlled, and data acquired, through Tiab digital controllers (v3.19.136.6).

Dislocation analysis was conducted with a JEOL JEM-2100F TEM/STEM (200 kV accelerating voltage). 0.5-mm-thick discs were cut from the gauge sections of the failed samples, both perpendicular and parallel to the loading direction, ground to 100−150 μm using SiC paper and electropolished using 3% perchloric acid, 57% methanol and 40% butan 1-ol in a Tenupol at −40 °C and 24 V. Dislocation analysis was carried out on foils for direct comparison with DDP results.

Transmission Kikuchi Diffraction (TKD) was carried out on TEM foils to identify grain pairs of interest for dislocation analysis. Data were collected on the Zeiss Sigma300 field emission gun scanning electron microscope (FEG-SEM) with an accelerating voltage of 30 kV, working distance of 3 mm and with the sample in the TKD holder normal to the electron beam. High-resolution TKD patterns were obtained in regions of samples (parallel and perpendicular to the loading direction), which showed high dislocation activity covering a hard−soft grain pair in a Zeiss Merlin FEG SEM equipped with a Bruker on-axis Optimus detector^55,56^. The Kikuchi patterns were further subdivided into 20 regions of interest (selected to ignore the area in the centre of the pattern resulting from the direct electron beam) and cross-correlated with reference patterns selected in a region away from any significant interest in the image map as described in refs. ^57,58^ in order to calculate the residual stress. The image and patterns were obtained at a 30 kV acceleration voltage and a probe current of 2 nA.

**Fig. 11:**
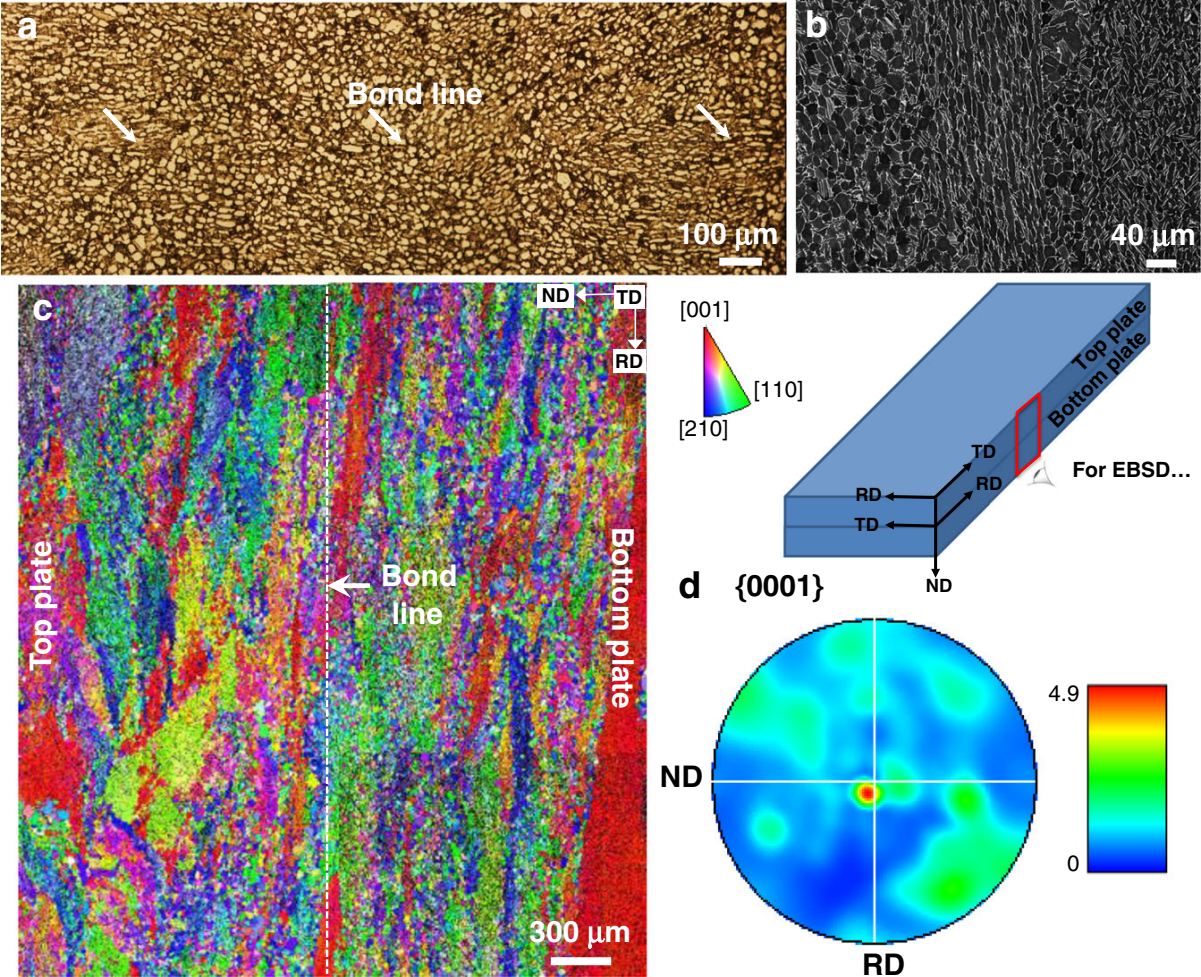
Experimental hard/soft bonded macrozone samples. **a** Optical micrograph showing the bond line in the deeply etched Ti-834 sandwich sample. **b** Back scattered electron image showing the microstructure of the alloy with primary alpha in transformed beta. **c** Large area EBSD scan showing the macrozone texture in the alloy relative to the bottom plate and **d** (0001) pole figure of *α* phase obtained from the large area scan in (**c**).

The optical micrograph in Fig. 11a shows the bond line on a deeply etched sample. A typical bimodal microstructure with primary alpha phase (*α*_p_) surrounded by transformed *β* is shown in Fig. 11b. Dark secondary alpha (*α*_s_) platelets can also be observed in the retained *β*. Figure 11c shows macrozones present in the alloy with ‘hard’ [0002]||RD macrozones (coloured red) adjacent to a ‘soft’ [0002]⊥RD macrozones (coloured green-blue). The macrozones are inherited from rolling of the prior beta grains and can be thought of as elongated flattened ellipsoids. The overall texture obtained from this large area EBSD scan showed a strong (0001) texture, Fig. 11d.

The two-dimensional DDP framework employed was firstly established in ref. ^29^. A temperature-coupled DDP (TDDP) approach incorporating thermal strains, thermally activated dislocation escape from obstacles, and temperature-dependent material properties^38^ has recently been developed for titanium alloys under thermomechanical conditions. The key features of the formulations are summarised here.

Dislocations nucleate from Frank-Read sources with density *ρ*_nuc_ in an initially dislocation-free specimen, where the resolved shear stress exerted on the source exceeds its strength *τ*_nuc_ for a period *t*_nuc_. A dislocation, once nucleated, glides along a predefined slip plane with velocity *v*_dis_ governed by a linear mobility rule:

1 $$v_{{\mathrm{{dis}}}} = \frac{{\tau b}}{B},$$

where *τ* is the resolved shear stress, *b* the magnitude of the Burgers vector and *B* a temperature-dependent drag coefficient. Mobile dislocations are pinned when encountering obstacles representing imperfections, forest dislocations and small precipitates, etc., which are randomly populated with a density *ρ*_obs_. Dislocations are released from such an obstacle by thermal activation when a residence time *t*_obs_ is achieved^35^:

2 $$\frac{1}{{t_{{\mathrm{{obs}}}}}} = {\mathrm{{\Gamma}}} = \frac{{\nu _{\mathrm{{D}}}b}}{{l_{{\mathrm{{obs}}}}}}{\mathrm{exp}}\left( { - \frac{{{\mathrm{{\Delta}}}H}}{{k\theta }}} \right){\mathrm{sinh}}\left( {\frac{{\tau {\mathrm{{\Delta}}}V}}{{k\theta }}} \right),$$

where *ν*_D_ is the Debye frequency, *l*_obs_ the average obstacle spacing ($l_{{\mathrm{{obs}}}} = 1/\sqrt {\rho _{{\mathrm{{obs}}}}}$), Δ*H* the activation energy, Δ*V* the activation volume, *k* the Boltzmann constant and *θ* is the temperature. Thermally activated dislocation escape events are hence governed by the two independent material properties, Δ*H* and Δ*V*.

## Supplementary information

Peer Review File

## Data Availability

The authors confirm that all relevant data are available upon reasonable request to the authors.
