# Supplementary material for: Predicting dwell fatigue life in titanium alloys using modelling and experiment
Source: Nat Commun. 2020 Nov 17;11:5868. doi: 10.1038/s41467-020-19470-w (PMC7672227; doi:10.1038/s41467-020-19470-w)
Supplement: Supplementary file 1 — Peer Review File [file 41467_2020_19470_MOESM1_ESM.pdf]

REVIEWER COMMENTS

Reviewer #1 (Remarks to the Author):

Please see the attached document for better clarity.

In this paper, the authors employ a combination of transmission electron microscopy (TEM), high-resolution electron backscatter diffraction (HR-EBSD), and discrete dislocation plasticity (DDP) modeling, to study the underlying mechanism of dwell fatigue in Titanium Alloy, i.e. Ti-834. Both experimental samples and numerical models are created containing a hypothesized worst-case microstructural feature, i.e. adjacent soft-hard crystallographic regions, to be investigated. The authors thus attempt to avoid the "sampling problem" which occurs in replicating large scale component response with small-scale laboratory specimens. Fatigue tests are conducted with three loading types, including two dwell (60s) loading with different stress levels and a thermal alleviation test at lower stress. The evolution of peak strain from each test is compared to DDP model results, and a thermal alleviation effect is demonstrated. DDP model calculations of dislocation activity near soft-hard grain boundaries are compared to TEM images and HR-EBSD measurements, showing good overall agreement.
The authors use the DDP model to estimate cycles to crack initiation under dwell and cyclic loading conditions. An extrapolation method is used to approximate the evolution of maximum stress in the hard grain and estimate cycles to fatigue crack initiation. Model results are then compared to experimental S-N curves.
The authors are advised to address the following comments:
Major comments
1. The authors attempt to address the sampling problem through micromechanical modeling of a crystallographic "worst-case" condition. The 2D microscopic model (10 um x 10 um) with a single "worst-case" grain-pair aims to represent a macroscopic experimental specimen (9 mm diameter x 36 mm length) specially processed to contain many crystallographic zones with the worst-case orientations. While soft-hard features exist in both model and the specimen, it should be noted that the volume of the experimental specimen is still orders of magnitude larger than the computational model, and potentially contains ~10,000's of distinct macrozones, based on Figure 11 and specimen dimensions.
i. An important aspect of the sampling problem that is neglected in the current work is the statistical quantification of the "worst-case" conditions in relation to the volume of the material. The volume of the model is Dwell-critical worst case arises from a probabilistic combination of both microstructural and mechanical states in a given volume of material. For example, extreme value theories have been employed to account for statistical size effects, e.g. in McDowell and Dunne, IJF 2010 and Ozturk et. al, Scripta 2017, Ozturk et. al, JMPS 2019 to successfully reproduce S-N curves, as well as fatigue life, scatter arising from microstructural variability.
ii. Another critical aspect of the sampling problem is the variation of the fatigue-driving mechanical fields over the structural volume, and their representation in polycrystalline models as boundary conditions. Such variations may arise from the structural geometry as addressed in Cuddihy et. al, IJF 2017. Another source of spatial variation is the heterogeneity of the microstructure, discussed in Ozturk et. al, JMPS 2019. The polycrystalline boundary conditions also evolve temporally over the course of fatigue cycles and deviate from the loads externally applied to test specimens.
However, the current work employs static, effective boundary conditions on a single, 2-dimensional polycrystalline model, and thus falls short of fully addressing the complexities of the sampling problem. This needs to be addressed.
2. Section 2.3.5: There are contradictions between the text in Section 2.3.5 and the model predictions plotted as S-N points in Fig 9d.

The text states: "Application of the same failure criterion using the DDP analysis to Loading Type Y (…) would not result in failure until ~105 cycles." This model outcome is expected, based on the slow rates of dislocation glide/pile-up, local stress rise etc. under load type Y, as presented in Figures 8 and 9b,c.

However, the S-N plot shows that the model-predicted life for LCDF at 675 MPa (Type Y) is ~11,000 cycles, which contradicts the text and the analyses presented.

Moreover, based on the text, if the criterion does not predict failure for LCDF (60 s hold) at 675 MPa until ~105 cycles, then it also may not predict failure for LCF (1 s hold) at the same load level (NLCF > NLCDF due to dwell effect). Therefore, the model-predicted life for LCF at 675 MPa, shown as ~20,000 cycles on the S-N plot, is also in contradiction with the text.

3. In Section 2.3.5, an extrapolation method is applied to the cyclic evolution of maximum basal stress to estimate the cycles to microscopic crack initiation. While the computational limitations of the problem are apparent, extrapolation methods should be used with caution and their pitfalls should be addressed before drawing conclusions from results. A common concern is whether rates have truly "stabilized" by the end of the computational stage (here, taken as 100 cycles), especially considering the continuously-evolving hardening mechanisms (e.g. dislocation interactions and back-stress effects) in polycrystalline materials under sustained stress-controlled loading. In fact, experimental data given in Figure 1 supports this concern as the rate of strain accumulation continues to change after the 100th cycle. Even if rates of output quantities seem to reach a steady-state for a small number of cycles, when studied over a cycle-scale of a few orders of magnitude (i.e. relevant to fatigue analyses) evolution is often observed to continue. Based on the reviewer's experience, extrapolation methods tend to grossly under-estimate life, especially for the low-stress/high-cycle cases which require extrapolations over a few orders of magnitude in the cycle domain. This would drastically skew the trend of the S-N curves in the high cycle/low-stress regime. In its current form, the accuracy of model-based results in Figure 9d is highly questionable. To provide a convincing analysis, the authors might want to consider the following:

i. adding a plot to Figure 9 to visualize the extrapolation procedure for the 6 tests, e.g. showing computed cyclic evolution of the quantities and their extrapolation lines intersecting σc=1200 MPa. (Note: using a logarithmic scale for cycles might improve the accuracy of the extrapolation process)
ii. validating the extrapolation method for at least one of the tests by running the DDP simulation beyond 100 cycles (at least an order of magnitude, e.g. up to ~1000 cycles), quantifying the error in the extrapolated quantity (ΔS=S-S*) and the corresponding error in the predicted number of cycles (ΔN=ΔS / [dS/dN]).
4. Section 2.3.5: The authors should address the difference between crack initiation life (the output of the model) and total fatigue life of large-scale specimens (experimental data). The DDP model output pertains to the event of stress exceeding the fracture capacity of a single microscopic location, i.e. microscopic crack initiation. However, experimental lives contain additional time spent during crack propagation, which takes place before the experimental observation of a macroscopic crack or specimen failure. Short and long crack propagation processes have distinct microstructure-sensitive mechanisms, and the latter is also influenced by the specimen geometry. It is understood that these are not subjects of the current modeling study, however, the results are directly compared to experimental lives in Fig 9 to support the validity of current modeling approach.
i. The distinction between the events of specimen failure (experimental data) and microscopic crack initiation (model output) should be acknowledged in the text.
ii. Can the authors justify making direct comparisons between the two different types of events in Figure 9d? Is it possible to transform between cycles of failure and initiation, e.g. by approximating propagation times, to achieve reasonable comparisons? In its current form, the proposed microscopic (DDP) model is potentially over-estimating crack initiation lives, as the true initiation lives in the material has to be earlier than the total fatigue lives plotted in Figure 9d.
iii. Based on the above points, perhaps the comparisons in Fig 9d are better interpreted as model successfully capturing the trends of the S-N response (assuming comment #3 is addressed), rather than capturing total failure lives with remarkable success, as claimed in the last paragraph of Section 2.3.5.
Minor comments, typos:
1. There are inconsistencies in the calculations of load levels relative to the yield stress. Please correct or clarify:
Section 2.1 defines peak stresses for type X and Y as 740 MPa and 675 MPa, and the yield stress as yield as 845MPa. These correspond to relative peak stresses of
- 0.88σY for type X
- 0.80σY for type Y

However,
- Section 3: refers to the peak stress of type X as $0.82\sigma Y$.
- Section 3: refers to the peak stress of type Y as $0.75\sigma Y$.
- Section 2.3.5: refers to the peak stress of type X as $0.90\sigma Y$.

2. Figure 4 / loading type Y: It would be helpful for the reader to know the number of load cycles this sample has gone through. Was this sample also loaded to failure?
3. Figure 3 caption: "Fig.5(c)" -> "Fig.3(c)"
4. Section 2.3.4, the "thermal alleviation" effect: Although the plot in Figure 8 clearly demonstrates an effect, the underlying mechanism is not obvious to the reviewer. It is counter-intuitive that an added stage of unidirectional loading at 350°C would un-do the plastic strain accumulated during the primary hold stage of a load cycle, considering:
i. The loading during 350°C period is along the same direction as the primary hold period (there is no macroscopic load reversal)
ii. Based on the temperature dependence of B and Γ in equations (1,2), one would expect an increased propensity for dislocation nucleation and glide during the high-temperature stage, but not necessarily a reversal of previously accumulated plastic strain
As the behavior is very interesting, it will improve the paper's impact if the authors could explain the underlying mechanism in more detail. The behavior does not seem to be directly related to the phenomenon explained in aspect #2 in page 235 of Zheng et. al, IJP 2018.
General inquiry: would a similar thermal alleviation also occur if the peak stress of the primary hold was 740 MPa?
5. Section 2.3.1: In the polycrystalline model for Ti-834, the soft grains, i.e. G3, G4, G6 are all at the free surface, which will induce an artificial boundary effect that is expected to bias the results. The free surfaces in micromechanical models may result in over-estimation of plastic accumulation, in comparison to a sub-surface polycrystalline domain (e.g., see Ozturk et al. JMPS 2019). Soft-hard grain pairs completely embedded and away from boundary might better represent the conditions of the problem.
6. Section 2.1: "dwell fatigue with at 740 MPa" -> "dwell fatigue at 740 MPa"
7. Please note that the polycrystalline microstructure in Figure 7c is a vertical mirror-reflection in comparison to those in Figures 7a and 7e – is this intentional? (does not necessarily require to be corrected).

Reviewer #2 (Remarks to the Author):

This is a good piece of work that is compromised by the writing, even accounting for (though perhaps as a consequence of) the Nature Communications house style.

There are three ways in which the key microstructural feature is described: appropriately orientated/badly orientated; hard/soft grains (macrozones); and 'bad metal sandwich'. Only the hard/soft grain concept is described in any depth and I suggest sticking with this as the sole descriptor. 'Appropriately orientated' and 'badly orientated' grains are not really explained. The term 'bad metal sandwich' is only introduced and used in the discussion and seems superfluous. I suggest that the crystallography of titanium is briefly explained, and how slip in HCP metals is governed by the different CRSSs of the slip systems and orientation relative to the loading direction. Without this the concept of hard and soft orientations is not obvious.

The structure should lead the reader to what should by this point be the obvious connection between the orientations of neighbouring grains / macrozones and the hypothesis to be tested. At the moment this is lost.

This could be an influential paper. I feel that the connection between the microstructure sampling in lab-scale and failure in the full scale could be strengthened. It is suggested in the Introduction that failure in the lab at higher fractions of the yield stress "is an artefact of the stressed volume of material subjected to testing and the likelihood of a test piece containing the critical microstructural feature." I do think that this has been demonstrated, so it might again be the sequence of events that needs to be re-ordered. Is there a probability of finding the defect that could be calculated? And do we never see lab tests that match large

scale tests? The sentence above suggests that we should sometimes observe this.

The rolling frame of reference appears to be inconsistent in figure 10. In the first half of the schematic the macrozones are normal to RD, whereas in the second half they are parallel to RD in both the upper and lower cut portions.

Being picky, in the method, the plane of the interface could be aligned in two ways to the loading direction, each of which would give equivalent results. However, it would be worth being clear about the orientation of the fatigue sample loading direction relative to the sample. I infer that this is parallel to RD of the bottom plate in figure 11, but this may not be the case. Also for clarity, the rolling frame of reference used in 11c and 11d is only relative to the bottom plate (I think).

Editorial suggestions:
I found the first sentence of the introduction somewhat oddly placed. I appreciate that it is there to provide context, but in order to do so I suggest a little more context, and a reference. Something along the lines of, "These accidents spurred research into the phenomenon and the use of fatigue resistance as a fundamental design parameter..."

The second paragraph could just focus on the phenomenon of dwell fatigue.

The final paragraph of the introduction transitions from its first sentence which reports frequent differences between lab tests and component tests (and which should be referenced) to the content of the current paper by way of an assertion that is supportable by the research in the paper, but not at this point in the paper. I suggest that the first sentence is moved to the paragraph discussing dwell fatigue and the linking sentence is rewritten slightly and coupled with the previous paragraph. Otherwise this would more usually be in the discussion of the current work.

Minor amendments
The second paragraph of the introduction contains a typo in its first line: "alloysd"
First paragraph of the results contains an error: "dwell fatigue with at 740 MPa"

Reviewer #3 (Remarks to the Author):

Ncomm review

The authors have performed a detailed analysis of the role of crystal orientation, stress level, and thermal excursions on strain accumulation. The work was of high quality and was both clearly and logically presented. I recommend that this work be published, but would urge the authors to address the following minor comments and request a mandatory discussion of the discrepancies between the authors approach and relevant published experimental data that has been omitted in the present work:

Minor comments:
— Top of pg 2: the hard grain unequivocally does NOT fracture one the basal plane. There have been reports of this since the 70's with more recent results (past 15 years) utilizing electron backscatter diffraction and also site-specific foil extraction via TEM. Our community needs to stop propagating this inaccuracy. It is a subtle detail, but it is important as it distinguishes dwell fatigue from cyclic fatigue. The fracture plane is more accurately described as an irrational {hail} plane inclined 10~20 deg. from (0001). References [3], [14], and [42] all discuss this, and there are other papers not cited here that show this clearly as well.

—Reference. [13] is irrelevant to this entire work — it deals with nucleation under continuous cycling in investment cast titanium; this results in a very different nucleation mechanism than is observed under dwell fatigue. The reviewer thinks the authors of this paper, perhaps, meant to cite A.L. Pilchak and J.C. Williams, Metallurgical and Materials Transactions A 42(4):1000-1027. This paper has detailed discussion of hard/soft

crystallography and its implication on dwell fatigue. It also clearly shows non-basal facet formation in the hard grain. The relevance of this work to the present manuscript is elaborated upon below.

— There is a mistake in fig 3 caption (it refers to 5(C))

— (pg 12) There was a large gap of work missed between Hasija (2003) and Zheng (2016, 2018) papers. Many others between these papers show exactly the same results that are attributed to Zheng, including Fig 7 in the Hasija paper, in fact.

— Figure 5(c) is confusing; If the crystal orientation is different, why does the schematic look the same? I also do not understand why you would restrict slip. As discussed below, other experiments have observed prism, basal a. and pyramidal <c+a> slip active in the 'hard grain' at the nucleation site of other near-alpha alloys.

Primary concern:
The authors have commented that TEM studies related to dwell fatigue are rare, cited their own work, but have overlooked at least four earlier contributions to the literature (& there are a few others that I haven't listed here), viz.

Technical Reports from The Ohio State Universities Program from the Federal Aviation Administration:
http://www.tc.faa.gov/its/worldpac/techrpt/ar0624.pdf
and
http://www.tc.faa.gov/its/worldpac/techrpt/tc17-57.pdf
As well as:
Pilchak, Brandes, Williams, and Williams: in Proc. World Conf. on Titanium, L. Zhou, H. Xhang, Y. Lu, D. Xu, eds., Science Press, Beijing, 2011, pp. 993-997.
And the work of Gerland et al.,
https://www.sciencedirect.com/science/article/abs/pii/S0921509308013488?via=ihub

The reviewer's only concern with the present paper is that there is no evidence that the specific grain orientations that the authors have chosen to analyze have ever been implicated in the nucleation of dwell fatigue cracks in laboratory specimens or failed components. Two of the three works cited above show experimental evidence of a different set of grain orientations at nucleation sites. Specifically, nucleation occurred on basal planes that were inclined to the stress axis and then immediately propagated into hard-oriented grains with the c-axis near parallel to the stress axis. In this situation, the "soft grain" would then be the one deforming by basal slip and the grain with [c] near parallel to the stress axis would be the hard grain. It was shown that pileups of basal slip bands in the soft grain, and not prismatic <a> slip, preceded crack nucleation. Similar orientations were implicated in the crack nucleation process by other authors (https://onlinelibrary.wiley.com/doi/abs/10.1111/j.1365-2818.2009.03141.x) and also (https://www.sciencedirect.com/science/article/pii/S1359646220301767?via=ihub). Moreover, while the reviewer generally agrees that basal slip in the hard grain can assist the facet formation process, there is experimental evidence that it is necessary. In fact, the World Ti Conf paper referenced above demonstrates a case where faceted propagation through a hard grain without any assistance of basal slip.

That being said, the authors have demonstrated through very careful TEM work and simulation that similar pileups can occur in prism-slip-oriented soft grains and stimulate basal slip. What they haven't done is show that this pair of grains is important with respect to nucleation. To the reviewers knowledge, The work of Sackett et al. [https://www.sciencedirect.com/science/article/abs/pii/S014211230600363X] on VERY coarse grain, beta-annealed IMI-685 is only one case of prism-slip-oriented soft grain leading to dwell fatigue crack nucleation. There were only a handful of grains in the sample, so the present authors will recognize that this work did not include all statistical combinations of hard/soft grain types, and hence this study should not be taken as evidence of prism <a> slip in a soft grain being implicated in nucleation in conventionally processed alpha+beta processed microstructures.

The work in http://www.tc.faa.gov/its/worldpac/techrpt/tc17-57.pdf
was performed at room temperature shows no such strong pileups forming on pyramidal <a> systems. Instead, secondary prismatic <a> systems were able to operate to relieve the stress of the pileup. In contrast, basal slip seemed to produce the most damaging condition because there are no secondary easy <a>-type slip systems to relieve pileup stresses. This enables strong basal <a> pileups to for resulting in both <c+a> pyramidal dislocations in the soft grain (to accommodate the increasing lattice rotation near the grain boundary due to the pileup) and also stimulating wavy <c+a> glide in the hard grain that was similar to what has been observed in two different TEM foils extracted from fracture facets formed in the hard grain of a soft/hard combination that nucleated.

Nevertheless, the present results are novel observations that provide new insights which cannot be ignored. On the same token, the results of the prior investigations cannot be overlooked and needs to be addressed in the context of this study. The past studies have, however, provided direct TEM evidence of the "basal soft / hard" grain combination at the initiation site of two cracks, and this has been confirmed additional times using electron backscatter diffraction. The authors of the present manuscript should provide direct evidence from fractography + EBSD or fractography + TEM foil extraction from the fracture surface that the prism-soft / hard combinations give rise to crack nucleation in an experimental sample configuration.

As a final note — the paper and this review have largely discussed a two-grain idealized model. Of course a given 'hard grain' has more than one neighbor. While the past studies contended that the "basal soft / hard" combination had the proper configuration to give rise to a crack, it is entirely possible that another grain, not captured in the foil and not readily analyzed by diffraction directly from the fracture surface (because prism-<a>-deforming grains do not form facets). had deformed by mechanisms similar to those described in the present paper and added an additional degree of load shedding onto the hard grain and thus may have been instrumental in the nucleation process.

To summarize, I believe this paper is worthy of acceptance in N Comm, but the authors need to (preferably) show clearly that their 'hard/soft' grain orientations actually result in crack nucleation and/or add considerable discussion about their work in the context of what has been reported for nearly 10 years in the literature that contradicts their choice of "soft" grain orientation. While that specific combination has been computationally identified as particularly bad in this study (https://royalsocietypublishing.org/doi/abs/10.1098/rspa.2007.1833), it did not include experimental validation, nor did it assess "basal soft" oriented grains either so this work is not sufficient to address the comment above.

The area of dwell fatigue is an extremely important issue and represents a safety concern for civil aviation. It has resulted in numerous unconfined engine failures and hence is a very important subject. This community is currently struggling to understand the role of temperature on the dwell fatigue debit as well as therm alleviation, and this body of works presents new data and insights. Consequently, I believe the work will be of great interest to the community.

I appreciate the opportunity to review this work and am happy to contribute to a transparent review process.

Very respectfully,
Adam Pilchak
US Air Force Research Laboratory (AFRL / RXCM)

Reviewer #1

(Remarks to the Author):

Please see the attached document for better clarity.

In this paper, the authors employ a combination of transmission electron microscopy (TEM), high-resolution electron backscatter diffraction (HR-EBSD), and discrete dislocation plasticity (DDP) modeling, to study the underlying mechanism of dwell fatigue in Titanium Alloy, i.e. Ti-834. Both experimental samples and numerical models are created containing a hypothesized worst-case microstructural feature, i.e. adjacent soft-hard crystallographic regions, to be investigated. The authors thus attempt to avoid the "sampling problem" which occurs in replicating large scale component response with small-scale laboratory specimens.

Fatigue tests are conducted with three loading types, including two dwell (60s) loading with different stress levels and a thermal alleviation test at lower stress. The evolution of peak strain from each test is compared to DDP model results, and a thermal alleviation effect is demonstrated. DDP model calculations of dislocation activity near soft-hard grain boundaries are compared to TEM images and HR-EBSD measurements, showing good overall agreement.

The authors use the DDP model to estimate cycles to crack initiation under dwell and cyclic loading conditions. An extrapolation method is used to approximate the evolution of maximum stress in the hard grain and estimate cycles to fatigue crack initiation. Model results are then compared to experimental S-N curves.

The authors are advised to address the following comments:

Major comments

Comment 1

1. The authors attempt to address the sampling problem through micromechanical modeling of a crystallographic "worst-case" condition. The 2D microscopic model (10 um x 10 um) with a single "worst-case" grain-pair aims to represent a macroscopic experimental specimen (9 mm diameter x 36 mm length) specially processed to contain many crystallographic zones with the worst-case orientations. While soft-hard features exist in both model and the specimen, it should be noted that the volume of the experimental specimen is still orders of magnitude larger than the computational model, and potentially contains ~10,000's of distinct macrozones, based on Figure 11 and specimen dimensions.

i. An important aspect of the sampling problem that is neglected in the current work is the statistical quantification of the "worst-case" conditions in relation to the volume of the material. The volume of the model is Dwell-critical worst case arises from a probabilistic combination of both microstructural and mechanical states in a given volume of material. For example, extreme value theories have been employed to account for statistical size effects, e.g. in McDowell and Dunne, IJF 2010 and Ozturk et. al, Scripta 2017, Ozturk et. al, JMPS 2019 to successfully reproduce S-N curves, as well as fatigue life, scatter arising from microstructural variability.

ii. Another critical aspect of the sampling problem is the variation of the fatigue-driving mechanical fields over the structural volume, and their representation in polycrystalline models as boundary conditions. Such variations may arise from the structural geometry as addressed in Cuddihy et. al, IJF 2017. Another source of spatial variation is the heterogeneity of the microstructure, discussed in Ozturk et. al, JMPS 2019. The polycrystalline boundary conditions also evolve temporally over the course of fatigue cycles and deviate from the loads externally applied to test specimens.

However, the current work employs static, effective boundary conditions on a single, 2-dimensional polycrystalline model, and thus falls short of fully addressing the complexities of the sampling problem. This needs to be addressed.

Our response:

i: We agree with the referee that ultimately, the 'worst case' will be determined by microstructural and mechanical states – and hence extreme statistics. As the referee recognises, we have addressed what we hypothesise to be a key extreme of the microstructure (that of macrozones and their orientation combination to loading) and tested this hypothesis in laboratory tests and in a sub-model representation. We have included a

short statement on the importance of extreme statistics in connection with microstructure and referenced this accordingly in Page 1 of the revised manuscript.

ii: The sensitivity to the mechanical fields imposed on the polycrystalline RVE has now been investigated. While it has not been possible to examine the evolution of the local mechanical boundary conditions, the sensitivity to a range of differing, representative microstructural boundary conditions has been examined. It's noted that the initial loading cycle has enormous impact on microstructural key variables (slip evolution, dislocation density, residual stress) and that the variations developed in subsequent cycles are often much smaller (eg J Jiang (https://doi.org/10.1016/j.actamat.2013.08.027 and https://doi.org/10.1016/j.actamat.2015.04.031)). Two additional forms of applied stress variation near the top surface (indicated as D-D' in Figure 1(a) below in this response) of the polycrystal model are used during the stress hold in one cycle of loading Type X (σ_{peak}=740MPa), in order to represent possible microstructure-induced variations. The two other distributions of the stresses are shown in Figure 1(b), which includes sinusoidal and arbitrary spatial variations with a mean stress level 740MPa and amplitude of 120MPa. The local stress distribution near the soft-hard grain pair which is key to understand the load shedding phenomenon and fatigue life of Ti834 specimens, is shown in Figure 1(c) for the three stress distributions. Interestingly, even with variation amplitude of 120MPa, the sensitivity of the local stress distribution is observed to be determined by the remote average loading from the boundary. In the revised manuscript, a discussion of this has now been included in respect of Figure 8 (manuscript). In addition, the role of differing and various boundary conditions and microstructure variations have also been studied in other papers (eg see Appendix B of https://doi.org/10.1016/j.ijplas.2020.102753), and they are similarly shown to have limited impact. Therefore, the polycrystal RVE model and loading shown in the manuscript reasonably represents the key region of interest under the loading conditions in the lab tests. We have briefly discussed the boundary conditions, loading and sampling on Page 6 of the revised manuscript.

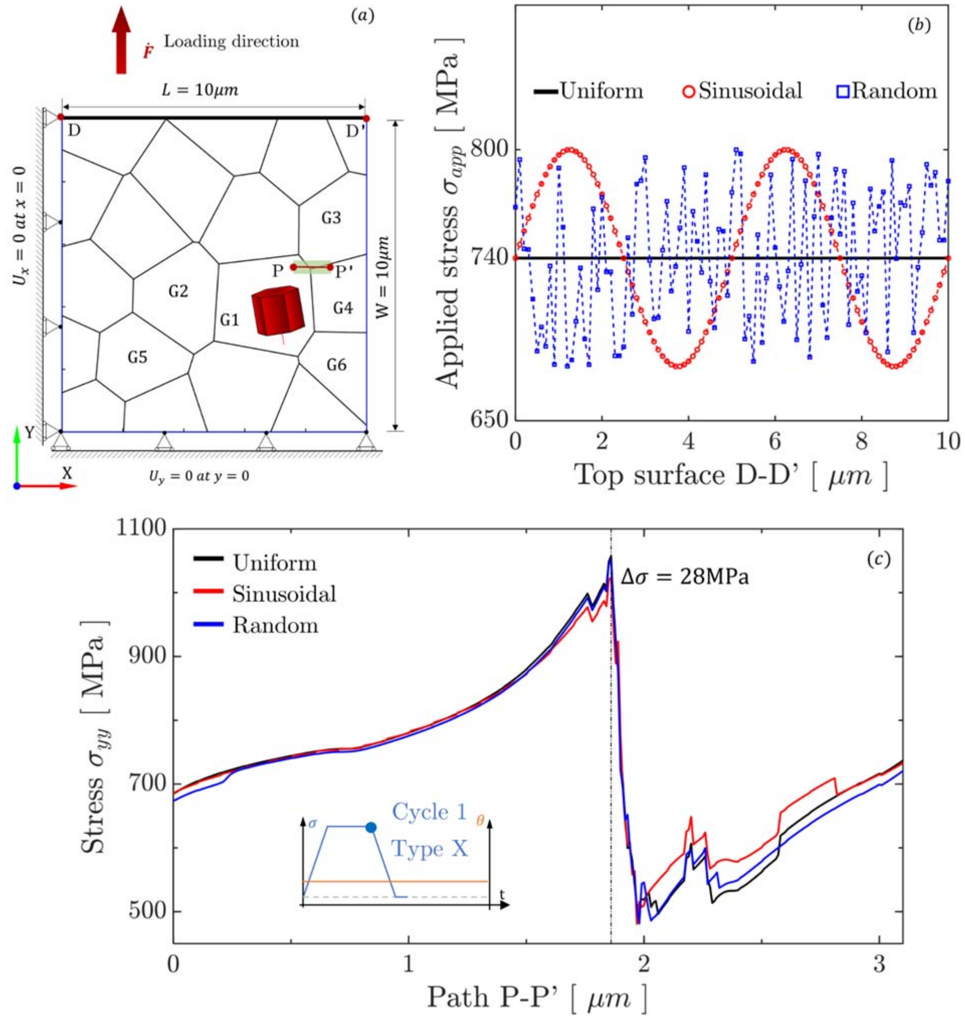

Figure 1. Effect of mechanical field variation on the local stress. (a) the DD polycrystal model showing loading boundary, (b) the three applied mechanical load types, and (c) the corresponding stress distribution along the path P-P' (indicated in (a)).

Comment 2

2. Section 2.3.5: There are contradictions between the text in Section 2.3.5 and the model predictions plotted as S-N points in Fig 9d.

The text states: "Application of the same failure criterion using the DDP analysis to Loading Type Y (…) would not result in failure until ~105 cycles." This model outcome is expected, based on the slow rates of dislocation glide/pile-up, local stress rise etc. under load type Y, as presented in Figures 8 and 9b,c.

However, the S-N plot shows that the model-predicted life for LCDF at 675 MPa (Type Y) is ~11,000 cycles, which contradicts the text and the analyses presented.

Moreover, based on the text, if the criterion does not predict failure for LCDF (60 s hold) at 675 MPa until ~105 cycles, then it also may not predict failure for LCF (1 s hold) at the same load level (NLCF > NLCDF due to dwell effect). Therefore, the model-predicted life for LCF at 675 MPa, shown as ~20,000 cycles on the S-N plot, is also in contradiction with the text.

Our response:

We apologize for a typographical error in the cycle number in the previous version of manuscript, and it has been corrected to 10^4 in the revised version such that the text is consistent with Fig 9(d).

Comment 3

3. In Section 2.3.5, an extrapolation method is applied to the cyclic evolution of maximum basal stress to estimate the cycles to microscopic crack initiation. While the computational limitations of the problem are apparent, extrapolation methods should be used with caution and their pitfalls should be addressed before drawing conclusions from results. A common concern is whether rates have truly "stabilized" by the end of the computational stage (here, taken as 100 cycles), especially considering the continuously-evolving hardening mechanisms (e.g. dislocation interactions and back-stress effects) in polycrystalline materials under sustained stress-controlled loading. In fact, experimental data given in Figure 1 supports this concern as the rate of strain accumulation continues to change after the 100th cycle. Even if rates of output quantities seem to reach a steady-state for a small number of cycles, when studied over a cycle-scale of a few orders of magnitude (i.e. relevant to fatigue analyses) evolution is often observed to continue. Based on the reviewer's experience, extrapolation methods tend to grossly under-estimate life, especially for the low-stress/high-cycle cases which require extrapolations over a few orders of magnitude in the cycle domain. This would drastically skew the trend of the S-N curves in the high cycle/low-stress regime. In its current form, the accuracy of model-based results in Figure 9d is highly questionable. To provide a convincing analysis, the authors might want to consider the following:

i. adding a plot to Figure 9 to visualize the extrapolation procedure for the 6 tests, e.g. showing computed cyclic evolution of the quantities and their extrapolation lines intersecting σc=1200 MPa. (Note: using a logarithmic scale for cycles might improve the accuracy of the extrapolation process)

ii. validating the extrapolation method for at least one of the tests by running the DDP simulation beyond 100 cycles (at least an order of magnitude, e.g. up to ~1000 cycles), quantifying the error in the extrapolated quantity (ΔS=S-S*) and the corresponding error in the predicted number of cycles (ΔN=ΔS / [dS/dN]).

Our response:

We thank the reviewer for their suggestion.

(i) We have added Figure 9(d) in the revised manuscript that details the DDP-predicted stresses over 100 calculated cycles, and in addition as requested, we have shown the extrapolation of the basal stress in the hard grain (G1 in Figure 5(a)) for the dwell fatigue simulations (Type X, Y and Z), which we have used to predict the lifetime of the samples.

(ii) The DDP-calculated cycles have been extended to 300 cycles for load Type Y (Ideally, more calculated cycles would be useful but became prohibitively CPU expensive to do). The extrapolation method has been validated by comparing the predicted basal stresses at 300 cycles with the extrapolated stresses (σ^{*}_{basal}). The error between the two stresses is calculated to be Δσ=21MPa or 2.7% at 300 cycles, which in turns gives an error in predicted number of failure cycles to be ΔN=ΔS / [dS/dN]= 430 (3-4%) cycles.

Modifications addressing this comment are at pp 11-12 of the revised manuscript (in red).

Comment 4

4. Section 2.3.5: The authors should address the difference between crack initiation life (the output of the model) and total fatigue life of large-scale specimens (experimental data). The DDP model output pertains to the event of stress exceeding the fracture capacity of a single microscopic location, i.e. microscopic crack initiation. However, experimental lives contain additional time spent during crack propagation, which takes place before the experimental observation of a macroscopic crack or specimen failure. Short and long crack propagation processes have distinct microstructure-sensitive mechanisms, and the latter is also influenced by the specimen geometry. It is understood that these are not subjects of the current modeling study, however, the results are directly compared to experimental lives in Fig 9 to support the validity of current modeling approach.

i. The distinction between the events of specimen failure (experimental data) and microscopic crack initiation (model output) should be acknowledged in the text.

ii. Can the authors justify making direct comparisons between the two different types of events in Figure 9d? Is it possible to transform between cycles of failure and initiation, e.g. by approximating propagation times, to achieve reasonable comparisons? In its current form, the proposed microscopic (DDP) model is potentially over-

estimating crack initiation lives, as the true initiation lives in the material has to be earlier than the total fatigue lives plotted in Figure 9d.

iii. Based on the above points, perhaps the comparisons in Fig 9d are better interpreted as model successfully capturing the trends of the S-N response (assuming comment #3 is addressed), rather than capturing total failure lives with remarkable success, as claimed in the last paragraph of Section 2.3.5.

Our response:

The referee is right to differentiate between cycles to nucleation and cycles to failure. However, experimental data (and one example is shown in Fig 1 for cycle X) for strain evolution shows that under stress-controlled loading, the approximately steady-state cyclic strain evolution late in life starts to change to higher strain accumulation per cycle, suggesting the progressive growth of defects. The strain rate upturn until failure occurs over ~10% of total life. It is noted that the bad metal sandwich sample (ie an adjacent hard and soft macrozone) is such that crack nucleation in the hard region is likely to be followed by rapid growth over a uniform region of crystallographic orientation, such that propagation time (cycles) is likely to be small compared to nucleation (reflecting the strain rate changes noted above). However, we have amended the text (in red) to include the point made by the referee and have changed it to indicate successfully capturing trends in the S-N curves.

Minor comments

Minor comments, typos:

1. There are inconsistencies in the calculations of load levels relative to the yield stress. Please correct or clarify:

Section 2.1 defines peak stresses for type X and Y as 740 MPa and 675 MPa, and the yield stress as yield as 845MPa. These correspond to relative peak stresses of

- $0.88\sigma Y$ for type X
- $0.80\sigma Y$ for type Y

However,

- Section 3: refers to the peak stress of type X as $0.82\sigma Y$.
- Section 3: refers to the peak stress of type Y as $0.75\sigma Y$.
- Section 2.3.5: refers to the peak stress of type X as $0.90\sigma Y$.

Our response:

We apologies for the inconsistency. These stress values have been all been corrected.

2. Figure 4 / loading type Y: It would be helpful for the reader to know the number of load cycles this sample has gone through. Was this sample also loaded to failure?

Our response:

It is stated as 2000 cycles on Page 11 of the manuscript.

3. Figure 3 caption: “Fig.5(c)” -> “Fig.3(c)”

Our response:

Thank you. The caption of Figure 3 has been corrected in Page 5 of the revised manuscript.

4. Section 2.3.4, the “thermal alleviation” effect: Although the plot in Figure 8 clearly demonstrates an effect, the underlying mechanism is not obvious to the reviewer. It is counter-intuitive that an added stage of unidirectional loading at 350°C would un-do the plastic strain accumulated during the primary hold stage of a load cycle, considering:

i. The loading during 350°C period is along the same direction as the primary hold period (there is no macroscopic load reversal)

ii. Based on the temperature dependence of B and Γ in equations (1,2), one would expect an increased propensity for dislocation nucleation and glide during the high-temperature stage, but not necessarily a reversal of previously accumulated plastic strain

As the behavior is very interesting, it will improve the paper's impact if the authors could explain the underlying mechanism in more detail. The behavior does not seem to be directly related to the phenomenon explained in aspect #2 in page 235 of Zheng et. al, IJP 2018.

General inquiry: would a similar thermal alleviation also occur if the peak stress of the primary hold was 740 MPa?

Our response:

The thermal elevation mechanisms for Ti834 have been systematically studied elsewhere (https://doi.org/10.1016/j.ijplas.2020.102753). Dislocations are found to reverse glide during the secondary stress hold period by the back stress that is accumulated at the primary stress hold period. The macroscopic strain, as a result of the reverse dislocation glide and dislocation structure relaxation, is decreased and the total creep strain in one load cycle is hence reduced. This creep strain reduction (possibly termed 'negative creep') is further enhanced at elevated temperatures as dislocation glide becomes more active (as pointed out by the reviewer).

5. Section 2.3.1: In the polycrystalline model for Ti-834, the soft grains, i.e. G3, G4, G6 are all at the free surface, which will induce an artificial boundary effect that is expected to bias the results. The free surfaces in micromechanical models may result in over-estimation of plastic accumulation, in comparison to a sub-surface polycrystalline domain (e.g., see Ozturk et al. JMPS 2019). Soft-hard grain pairs completely embedded and away from boundary might better represent the conditions of the problem.

Our response:

In fact, because of the plane strain condition in the DDP analysis, the front surface is not in fact a free surface and is much more representative of a deeply embedded microstructure. In addition, with respect to side boundaries, the effects have been investigated in other studies (eg please see Appendix in https://doi.org/10.1016/j.ijplas.2020.102753), where it has been found that the effects are small. In this study of the load shedding between soft grains (G3, G4 and G6) and hard grain (G1), the region of interest is placed at least 10% of the specimen width away from the side boundaries.

6. Section 2.1: "dwell fatigue with at 740 MPa" -> "dwell fatigue at 740 MPa"

Our response:

Thank you. This typo has been rectified.

7. Please note that the polycrystalline microstructure in Figure 7c is a vertical mirror-reflection in comparison to those in Figures 7a and 7e – is this intentional? (does not necessarily require to be corrected).

Our response:

Thank you. We indeed aim to provide a visually-close one-to-one comparison of the dislocation structure achieved in the DDP modelling and TEM experimental observation. We add a note (in red) to highlight the mirror-reflection in the caption of Figure 7 of the revised manuscript.

Reviewer #2

(Remarks to the Author):

This is a good piece of work that is compromised by the writing, even accounting for (though perhaps as a consequence of) the Nature Communications house style.

Comment 1

There are three ways in which the key microstructural feature is described: appropriately orientated/badly orientated; hard/soft grains (macrozones); and 'bad metal sandwich'. Only the hard/soft grain concept is described in any depth and I suggest sticking with this as the sole descriptor. 'Appropriately orientated' and 'badly orientated' grains are not really explained. The term 'bad metal sandwich' is only introduced and used in the discussion and seems superfluous. I suggest that the crystallography of titanium is briefly explained, and how slip in HCP metals is governed by the different CRSSs of the slip systems and orientation relative to the loading direction. Without this the concept of hard and soft orientations is not obvious.

Our response:

We thank the reviewer for this suggestion on the terminology and on providing the fundamental knowledge of HCP Ti alloys. We have unified the terminology throughout the revised manuscript preferring the referee's suggested hard/soft grains, but have maintained the brief statement in the introduction to explain macrozones because of their importance in the dwell literature, but now recognising that they also can take hard or soft overall orientations. Hence we think the terminology is now much more consistent throughout. We have also acted on the referee's comment to (briefly) refer to the slip system types, CRSSs and hard/soft in the context of loading direction, in the introduction.

Comment 2

The structure should lead the reader to what should by this point be the obvious connection between the orientations of neighbouring grains / macrozones and the hypothesis to be tested. At the moment this is lost.

This could be an influential paper. I feel that the connection between the microstructure sampling in lab-scale and failure in the full scale could be strengthened. It is suggested in the Introduction that failure in the lab at higher fractions of the yield stress "is an artefact of the stressed volume of material subjected to testing and the likelihood of a test piece containing the critical microstructural feature." I do think that this has been demonstrated, so it might again be the sequence of events that needs to be re-ordered. Is there a probability of finding the defect that could be calculated? And do we never see lab tests that match large scale tests? The sentence above suggests that we should sometimes observe this.

Our response:

The introduction has been reorganised to try to improve clarity such that now, with the referee's suggestions, we think the hypothesis of orientation combinations of macrozones driving dwell is now clearer. The lab-scale testing is discussed in the revised introduction and linked to the context of full-scale disc tests. In fact, the probability of defects (combinations of damaging orientations) have been estimated by our industrial collaborators for commercial alloys and this has driven the primary methodology reported – the manufacture of our 'bad metal sandwich' samples which facilitate laboratory testing of the 'worst case'. We hope this is now clearer.

Comment 3

The rolling frame of reference appears to be inconsistent in figure 10. In the first half of the schematic the macrozones are normal to RD, whereas in the second half they are parallel to RD in both the upper and lower cut portions.

Our response:

This has been corrected in Figure 10 of the revised manuscript, making the rolling frame of reference consistent.

Comment 4

Being picky, in the method, the plane of the interface could be aligned in two ways to the loading direction, each of which would give equivalent results. However, it would be worth being clear about the orientation of the fatigue sample loading direction relative to the sample. I infer that this is parallel to RD of the bottom plate in figure 11, but this may not be the case. Also for clarity, the rolling frame of reference used in 11c and 11d is only relative to the bottom plate (I think).

Our response:

The fatigue sample is loaded along the RD of the top plate. The rolling frame of reference in Fig. 11 c and d is relative to the bottom plate as inferred by the reviewer. This is now mentioned in the text in Sec. 4.1 and in the caption of Fig. 11 in the revised manuscript.

Editorial suggestions:

I found the first sentence of the introduction somewhat oddly placed. I appreciate that it is there to provide context, but in order to do so I suggest a little more context, and a reference. Something along the lines of, "These accidents spurred research into the phenomenon and the use of fatigue resistance as a fundamental design parameter..."

The second paragraph could just focus on the phenomenon of dwell fatigue.

The final paragraph of the introduction transitions from its first sentence which reports frequent differences between lab tests and component tests (and which should be referenced) to the content of the current paper by way of an assertion that is supportable by the research in the paper, but not at this point in the paper. I suggest that the first sentence is moved to the paragraph discussing dwell fatigue and the linking sentence is rewritten slightly and coupled with the previous paragraph. Otherwise this would more usually be in the discussion of the current work.

Our response:

We have modified the introduction by adding a little more context as suggested and providing a reference. We have amended the second paragraph to focus on dwell fatigue (by re-phrasing the statement about thermomechanical fatigue). We have also shifted the first statement of the last paragraph (old manuscript) to the dwell introduction and linked the modified last para with the previous para as suggested.

Minor amendments

The second paragraph of the introduction contains a typo in its first line: "alloysd"

First paragraph of the results contains an error: "dwell fatigue with at 740 MPa"

Our response:

Thank you. The typographical errors identified have been rectified in the revised manuscript.

Reviewer #3

(Remarks to the Author):

The authors have performed a detailed analysis of the role of crystal orientation, stress level, and thermal excursions on strain accumulation. The work was of high quality and was both clearly and logically presented. I recommend that this work be published, but would urge the authors to address the following minor comments and request a mandatory discussion of the discrepancies between the authors approach and relevant published experimental data that has been omitted in the present work:

Minor comments:

— Top of pg 2: the hard grain unequivocally does NOT fracture one the basal plane. There have been reports of this since the 70's with more recent results (past 15 years) utilizing electron backscatter diffraction and also site-specific foil extraction via TEM. Our community needs to stop propagating this inaccuracy. It is a subtle detail, but it is important as it distinguishes dwell fatigue from cyclic fatigue. The fracture plane is more accurately described as an irrational (Indurkar et al.) plane inclined 10~20 deg. from (0001). References [3], [14], and [42] all discuss this, and there are other papers not cited here that show this clearly as well.

Our response:

We thank the reviewer for the critical point, and the more accurate description has been used in Page 2 of the revised manuscript.

—Reference. [13] is irrelevant to this entire work — it deals with nucleation under continuous cycling in investment cast titanium; this results in a very different nucleation mechanism than is observed under dwell fatigue. The reviewer thinks the authors of this paper, perhaps, meant to cite A.L. Pilchak and J.C. Williams, Metallurgical and Materials Transactions A 42(4):1000-1027. This paper has detailed discussion of hard/soft crystallography and its implication on dwell fatigue. It also clearly shows non-basal facet formation in the hard grain. The relevance of this work to the present manuscript is elaborated upon below.

Our response:

Thanks for this comment, and the new reference has been updated in Page 1 of the revised manuscript.

— There is a mistake in fig 3 caption (it refers to 5(C))

Our response:

Thank you. The caption of Figure 3 has been corrected in Page 5 of the revised manuscript.

— (pg 12) There was a large gap of work missed between Hasija (2003) and Zheng (2016, 2018) papers. Many others between these papers show exactly the same results that are attributed to Zheng, including Fig 7 in the Hasija paper, in fact.

Our response:

We thank the reviewer for this suggestion on the reference. We have added several references that cover the gap, which is in Page 12 of the revised manuscript.

— Figure 5(c) is confusing; If the crystal orientation is different, why does the schematic look the same? I also do not understand why you would restrict slip. As discussed below, other experiments have observed prism, basal a. and pyramidal <c+a> slip active in the 'hard grain' at the nucleation site of other near-alpha alloys.

Our response:

The soft grain (b) shows the three in-plane prism slip systems; the hard grain (c) includes two in-(c-a) plane pyramidal systems, and a single in-(c-a) plane basal system. The hard grain c-axis in (c) is orientated at 11° to the loading direction to match the experiment, and we have properly reflected this now in Fig. 5. We confine the slip systems to 3 prism <a> types in the soft grains and 2 basal <a> plus 1 pyramidal <c+a> in the hard grain, as a condition imposed by the plane strain constraint in two-dimensional DD simulation (please see http://iopscience.iop.org/0965-0393/3/5/008) and its application in Ti crystals (please see http://dx.doi.org/10.1016/j.jmps.2016.08.008). This does reduce the representation of all possible (3D) slip activations but has the advantage that we are representing sub-surface behaviour that is also not too dissimilar to macrozone morphology.

Primary concern:

Comment 1

The authors have commented that TEM studies related to dwell fatigue are rare, cited their own work, but have overlooked at least four earlier contributions to the literature (& there are a few others that I haven't listed here), viz.

Technical Reports from The Ohio State Universities Program from the Federal Aviation Administration:

http://www.tc.faa.gov/its/worldpac/techrpt/ar0624.pdf and

http://www.tc.faa.gov/its/worldpac/techrpt/tc17-57.pdf

As well as: Pilchak, Brandes, Williams, and Williams: in Proc. World Conf. on Titanium, L. Zhou, H. Xhang, Y. Lu, D. Xu, eds., Science Press, Beijing, 2011, pp. 993-997. And the work of Gerland et al. https://www.sciencedirect.com/science/article/abs/pii/S0921509308013488?via=ihub

Our response:

We thank the referee and agree that the works mentioned by the reviewer have extensive TEM analysis on grain pairs under dwell fatigue conditions. These are now included in the Introduction (Page 2) of the revised manuscript. In our work, we have been able to show definitively that deformation during dwell fatigue does lead to prism dislocation pile-ups (not present in AR) at hard grains together with basal and pyramidal slip activation in the hard-orientated grain.

Comment 2

The reviewer's only concern with the present paper is that there is no evidence that the specific grain orientations that the authors have chosen to analyze have ever been implicated in the nucleation of dwell fatigue cracks in laboratory specimens or failed components. Two of the three works cited above show experimental evidence of a different set of grain orientations at nucleation sites. Specifically, nucleation occurred on basal planes that were inclined to the stress axis and then immediately propagated into hard-oriented grains with the c-axis near parallel to the stress axis. In this situation, the "soft grain" would then be the one deforming by basal slip and the grain with [c] near parallel to the stress axis would be the hard grain. It was shown that pileups of basal slip bands in the soft grain, and not prismatic slip, preceded crack nucleation. Similar orientations were implicated in the crack nucleation process by other authors

(https://onlinelibrary.wiley.com/doi/abs/10.1111/j.1365-2818.2009.03141.x) and also (https://www.sciencedirect.com/science/article/pii/S1359646220301767?via=ihub).

Moreover, while the reviewer generally agrees that basal slip in the hard grain can assist the facet formation process, there is experimental evidence that it is necessary. In fact, the World Ti Conf paper referenced above demonstrates a case where faceted propagation through a hard grain without any assistance of basal slip.

That being said, the authors have demonstrated through very careful TEM work and simulation that similar pileups can occur in prism-slip-oriented soft grains and stimulate basal slip. What they haven't done is show that this pair of grains is important with respect to nucleation. To the reviewers knowledge, The work of Sackett et al. [https://www.sciencedirect.com/science/article/abs/pii/S014211230600363X] on VERY coarse grain, beta-annealed IMI-685 is only one case of prism-slip-oriented soft grain leading to dwell fatigue crack nucleation. There were only a handful of grains in the sample, so the present authors will recognize that this work did not include all statistical combinations of hard/soft grain types, and hence this study should not be taken as evidence of prism slip in a soft grain being implicated in nucleation in conventionally processed alpha+beta processed microstructures.

Our response:

[Editorial note: One of us (DD) thanks the reviewer for pointing out the Sackett et al paper – I had seen it presented but had never found the actual paper.]

It is likely that crack nucleation on a particularly oriented grain pair under dwell fatigue depends on the surrounding microstructure and the loading conditions as well as the particular combination of orientations of the grain pair, and it varies from sample to sample. In this work we considered a grain pair with a maximum possible misorientation in such a way that the resistance to slip transmission across the boundary is maximised so that the large pile-up stress in the soft grain nucleates slip in the hard grain. It's a model study to explore the dislocation activities in a soft/hard grain pair which envisages a modified Stroh pile-up model to explain the crack nucleation

under dwell conditions. However, in this specific orientation combination, the hard grain basal normal is inclined to the loading direction by ~11° and hence is different to those considered in report tc17-57, but not completely different in character to the case of c-axis parallel to remote loading, for which there is the established link with crack nucleation/growth (referred to as secondary growth in report http://www.tc.faa.gov/its/worldpac/techrpt/ar0624.pdf). It's noted in this respect that even in the latter, a remote direct stress normal to a basal plane does not assure that the local max principal stress is also normal to the basal at the grain of interest. We have now noted our main objective in Sec. 2.2 (Page 3) of the revised manuscript.

We have done extensive TEM analysis on grain pairs with different crystallographic orientations under LCF and dwell fatigue which have been recently published in S Joseph et al., Int. J. Plast. 102743 (2020). doi:10.1016/j.ijplas.2020.102743. It is difficult to analyse grain pairs with different crystallographic orientations in the present study as we have applied multiple techniques to focus in some detail viz., DDP, HR-EBSD and TEM to understand the comprehensive behaviour of a single grain pair.

Comment 3

The work in http://www.tc.faa.gov/its/worldpac/techrpt/tc17-57.pdf was performed at room temperature shows no such strong pileups forming on pyramidal systems. Instead, secondary prismatic systems were able to operate to relieve the stress of the pileup. In contrast, basal slip seemed to produce the most damaging condition because there are no secondary easy -type slip systems to relieve pileup stresses. This enables strong basal pileups to for resulting in both <c+a> pyramidal dislocations in the soft grain (to accommodate the increasing lattice rotation near the grain boundary due to the pileup) and also stimulating wavy <c+a> glide in the hard grain that was similar to what has been observed in two different TEM foils extracted from fracture facets formed in the hard grain of a soft/hard combination that nucleated.

Nevertheless, the present results are novel observations that provide new insights which cannot be ignored. On the same token, the results of the prior investigations cannot be overlooked and needs to be addressed in the context of this study. The past studies have, however, provided direct TEM evidence of the "basal soft / hard" grain combination at the initiation site of two cracks, and this has been confirmed additional times using electron backscatter diffraction. The authors of the present manuscript should provide direct evidence from fractography + EBSD or fractography + TEM foil extraction from the fracture surface that the prism-soft / hard combinations give rise to crack nucleation in an experimental sample configuration.

Our response:

The <c+a> dislocation pile ups in the hard grain were observed since the hard grain in our case is well oriented for pyramidal slip. Further, the nucleation of basal dislocations in the hard grain was observed in places where the prism pile-ups in the soft grain hits the boundary. Hence, we believe that the pyramidal slip is due to the crystallographic orientation of the grain and the basal slip is due to load shedding. Further, it is difficult to compare the TEM observations in 'http://www.tc.faa.gov/its/worldpac/techrpt/tc17-57.pdf' to our study as the crystallographic orientations of grains investigated are different.

This study is probably too much to add into the present manuscript (Joseph and Dye, draft attached), but its rather too useful to just leave as supplementary information, so we have written it up as a separate letter. In the initiating grain we do in fact find basal slip bands, as the reviewer suggests. In contrast, in report http://www.tc.faa.gov/its/worldpac/techrpt/ar0624.pdf examples of soft (prism) – hard grain cracking. There are also other examples of soft (basal) – hard grain dwell cracking [Bache, IJF 19,1,1997; Woodfield et al, Ti95] and quite recent CP modelling work supports these observations in the sense that the basal stresses after load shedding are higher than those which arise with soft (prism) slip [Zhang et al, IJF, 113, 324-334, 2017].

Therefore the state of the observations in the literature seem to be that either basal or prism <a> slip occurs in the soft grain, depending on the specifics of the grain environment, notwithstanding the elegance of the argument around stress relief by secondary slip that the reviewer advances. In either case, the key point is that <a>-type slip occurs in the soft grain, that load shedding between the soft and hard grain is critical, that macrozones giving a larger effective slip length for slip bands to accumulate strain are important, and that the relative availability of other slip systems can be modified by temperature and exposure time to give the characteristic cold dwell fatigue response. i.e., we don't think that the debate over bas<a> vs pri<a> in the soft grain is critical to the validity of our main findings.

Therefore we have added a paragraph in p13 to discuss these points.

Comment 4

As a final note — the paper and this review have largely discussed a two-grain idealized model. Of course a given 'hard grain' has more than one neighbor. While the past studies contended that the "basal soft / hard" combination had the proper configuration to give rise to a crack, it is entirely possible that another grain, not captured in the foil and not readily analyzed by diffraction directly from the fracture surface (because prism--deforming grains do not form facets). had deformed by mechanisms similar to those described in the present paper and added an additional degree of load shedding onto the hard grain and thus may have been instrumental in the nucleation process.

To summarize, I believe this paper is worthy of acceptance in N Comm, but the authors need to (preferably) show clearly that their 'hard/soft' grain orientations actually result in crack nucleation and/or add considerable discussion about their work in the context of what has been reported for nearly 10 years in the literature that contradicts their choice of "soft" grain orientation. While that specific combination has been computationally identified as particularly bad in this study (https://royalsocietypublishing.org/doi/abs/10.1098/rspa.2007.1833), it did not include experimental validation, nor did it assess "basal soft" oriented grains either so this work is not sufficient to address the comment above.

Our response:

We also recognise the difficulty in understanding the driver of dwell faceting by focusing on a single neighbour, and that it may well be a combination of contributions from other neighbours which ultimately secure the facet formation. However, our hypothesis is to simplify and understand at least what the mechanisms taking place are in what we consider to be a close to worst-case scenario. That is, the controlled texture combination available to us from the 'bad metal sandwich' methodology developed. This paper reports our findings. However, subsequent TEM studies (Joseph and Dye) yet to be published and carried out on the very same soft (prism slip) – hard grain system studied here does in fact provide causal link to establish that the orientation combination selected does lead to facet formation (see above). Incidentally, this combination (considered in this paper) has always been the favoured hypothesis coming anecdotally from our industrial partners in the context of disc components (as opposed to lab samples), and has been reported to lead to dwell cracking for a number of examples in http://www.tc.faa.gov/its/worldpac/techrpt/ar0624.pdf. We would add to the discussion that there remain many problems in capturing the true conditions leading to in-service dwell fatigue through use of laboratory (as opposed to component) testing. One of these is simply the volume effect to ensure that the worst-case microstructure is present in the sample being tested. This is the basis for our hypothesis to test a near worst case crystal combination at macrozone/MTR length scales. Another major factor is magnitude of applied loading in lab tests; many tests are subjected to 0.9-0.95σ_y and higher stresses which are not representative of the stresses established in components, at least for one major business user. It's not definitively clear that any of the observations made at these elevated loads are capable of reproducing the mechanisms to be representative of dwell fatigue in components.

The referee rightly raises the issue of the selection of soft (prism) – hard combination over the alternative of a soft (basal) – hard. However, it is the case that we have investigated this using modelling in some detail (Zhang et al, 'Phase morphology, variants and crystallography of alloy microstructures in cold dwell fatigue', Intl, Jnl. Fatigue, 113, 324-334, 2017). This study investigates both soft grain creep evolution and stress redistribution (load shedding) on to hard grains for the two separate cases of soft (basal) and soft (prism) grains adjacent to a hard grain. This became meaningful to do because we had managed to extract out the differing intrinsic basal and prism slip system rate sensitivities from controlled micro-pillar tests (Jnl. Mech. Phys. Solids, 95, 393–410, 2016). The overall characteristics under dwell fatigue were found to be similar but interestingly, the soft (basal) case led to somewhat higher creep evolution and hence to higher stresses developing on the hard grain. This would support a number of the experimental observations made in the literature [eg Bache, IJF 19,1,1997; Woodfield et al, Ti95] but in our view, does not discount the possibility of facet formation from the soft (prism) – hard combination. Indeed, the results yet to be published by Joseph and Dye demonstrate this. A discussion of the relevant aspects of the above has now been included in the revised manuscript.

REVIEWER COMMENTS

Reviewer #3 (Remarks to the Author):

The authors have addressed all the issues. However, in my opinion, there are still two questions need to be revised before accepted.
1, According to figure 1d and e, the relatively large hysteresis still exists at VDS=6.5V after the forming process at 5.5 V. It seems contradictory to the results in Figure R2c and d with almost no hysteresis. Based on the forming mechanism about H2O adsorption, dissociative adsorption can be transited to molecular adsorption during the forming process. Therefore, the hysteresis at 6.5V may be contradictory to the known characteristic of molecular adsorption.

2, What is the value of the gate voltage used to read Ids in Figure 2b to j ? According to Figure R4, the linearity of the Ids can be improved if the optimized gate voltage was applied. So, whether the accuracy in Figure 4 will be raised furtherly under an optimized gate voltage?
